# Supplementary material for: Mobility-driven synthetic contact matrices as a scalable solution for real-time pandemic response modeling
Source: Nat Commun. 2026 Jan 27;17:1845. doi: 10.1038/s41467-026-68557-3 (PMC12920812; doi:10.1038/s41467-026-68557-3)
Supplement: Supplementary file 1 — Supplementary Information [file 41467_2026_68557_MOESM1_ESM.pdf]

SUPPLEMENTARY INFORMATION

Mobility-driven synthetic contact matrices as a scalable solution for real-time pandemic response modeling

Laura Di Domenico[1,+], Paolo Bosetti[2], Chiara E. Sabbatini[3,++], Lulla Opatowski[4,5], Vittoria Colizza[3,6,*]

[1]*Institute of Social and Preventive Medicine, University of Bern, Bern, Switzerland*
[2]*Mathematical Modelling of Infectious Diseases Unit, Institut Pasteur, Université Paris Cité, CNRS UMR 2000, Paris, France*
[3]*Sorbonne Université, INSERM, Pierre Louis Institute of Epidemiology and Public Health, Paris, France* [4]*Institut Pasteur, Epidemiology and Modelling of Antibiotic Evasion, Université Paris Cité, Paris, France*
[5]*UVSQ, INSERM, CESP, Anti-Infective Evasion and Pharmacoepidemiology Team, Université Paris-Saclay, Montigny-Le-Bretonneux, France*
[6]*Department of Biology, Georgetown University, Washington, District of Columbia, USA*

[+]Currently at Data Science Institute, Hasselt University, Hasselt, Belgium
[++]Currently at Santé publique France, French National Public Health Agency, Saint-Maurice, France

*corresponding author: vittoria.colizza@inserm.fr

Table of Contents

1. TRANSMISSION MODEL 3

1.1 STRUCTURE OF THE COMPARTMENTAL MODEL 3
1.2 MODEL PARAMETERS FOR THE WUHAN STRAIN 4

2. VARIANT-SPECIFIC PARAMETERS 5

2.1 GENERATION TIME 5
2.2 TRANSMISSION ADVANTAGE 5
2.3 HOSPITALIZATION RISK 5
2.4 SUSCEPTIBILITY 5

3. POPULATION IMMUNITY 6

3.1 INFECTION-INDUCED IMMUNITY 6
3.2 VACCINE EFFECTIVENESS 6
3.3 HYBRID IMMUNITY 7

4. PARAMETERIZATION OF CONTACT MATRICES 8

4.1 SCHOOL CALENDAR 8
4.2 CONSTRUCTION OF SYNTHETIC MATRICES 9
4.2.1 Modeling telework 9
4.2.2 Modeling partial or full school closure 10
4.2.3 Leisure and other activities 11
4.2.4 Avoidance of physical contacts 11
4.2.5 School holidays 11
4.2.6 Summary table 12
4.3 ADAPTATION AND INTEGRATION OF THE SOCIALCOV CONTACT MATRICES 13
4.4 MODELING THE IMPACT OF TESTING AND SELF-ISOLATION 13

5. METRICS USED TO COMPARE CONTACT MATRICES 14

5.1 AVERAGE CONNECTIVITY 14

5.2 Cosine similarity 14
5.3 Proportion of young connectivity 14
5.4 Assortativity index 14
5.5 Proportion of within-group contacts 15

6. INFERENCE FRAMEWORK 16

7. TRANSMISSION MODEL COMPARISON 19

7.1 Model fit 19
7.2 Correcting factor 20
7.3 Hospitalizations by age class 22
7.4 Attack rate 23

8. ADDITIONAL RESULTS ON COMPARISON OF CONTACT MATRICES 24

8.1 Comparison of mixing patterns 24
8.2 Correlation with normalcy and stringency index 24
8.3 Comparison with CoMix matrices 25

9. SENSITIVITY ANALYSES 28

9.1 Specification of empirical contact matrices 28
9.2 Relative susceptibility of young individuals 30
9.3 Calibration targets 31
9.4 Variations in the total number of contacts per age group 35
9.5 Variations in mixing patterns 37
9.6 ignoring avoidance of physical contacts 40

10. REFERENCES 43

1. TRANSMISSION MODEL

1.1 STRUCTURE OF THE COMPARTMENTAL MODEL

We used a stochastic age-stratified transmission model, accounting for two-strain dynamic (Wuhan-Alpha, Alpha-Delta, Delta-Omicron) and for vaccination. We used four age classes: children [0-10], adolescents [11-18], adults [19-64] and seniors (65+ years old). Disease progression in terms of compartments for one layer of the model is illustrated in **Fig. S1**. The complete model includes additional strata with analogous compartments to distinguish for age, vaccination status and waning, variant of current and prior infection.

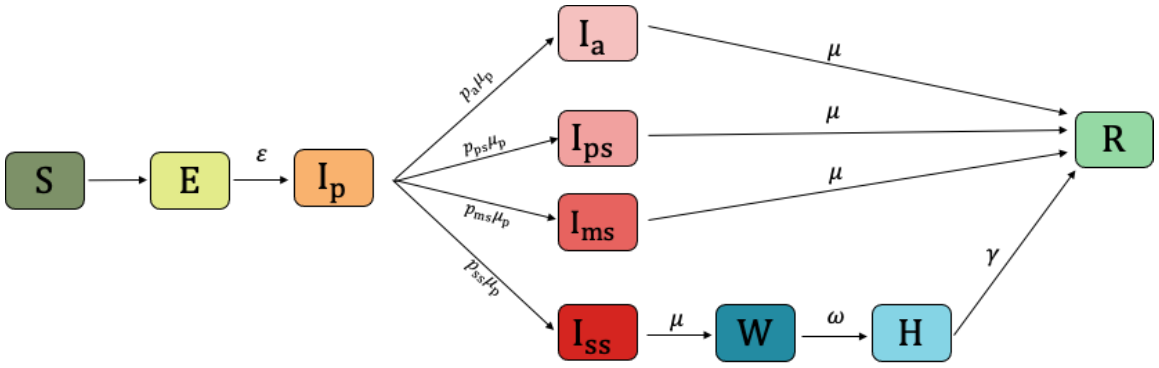

Figure S1. Scheme of the compartments of the transmission model. Individuals in E are infected but not infectious, while compartments I_p, I_a, I_{ps}, I_{ms}, I_{ss} can transmit the disease. S = susceptible; E = exposed; I_p = infectious in the prodromic phase (the length of time including E and I_p stages is the incubation period); I_a = asymptomatic infectious; I_{ps} = pauci-symptomatic infectious; I_{ms} = symptomatic infectious with mild symptoms; I_{ss} = symptomatic infectious with severe symptoms; W = severe case waiting to be admitted to hospital; H = severe case admitted to the hospital; R = removed.

In **Fig. S2**, we provide an illustrative scheme of the additional strata in the model.

We considered a two-strain history-based model, i.e. a model where we track infections with different strains by splitting all compartments into two, except for the compartment S for susceptibles. For the Wuhan-Alpha model and the Alpha-Delta model, we considered complete cross-immunity, i.e. upon infection with either one of the two strains, an infected individual eventually enters compartment R and becomes immune to the other strain (**Fig. S2a,b**). Initial condition for the R compartment in the Alpha-Delta model was informed from the final size of R compartment in the Wuhan-Alpha model. For the Delta-Omicron epidemic, we considered possible re-infection with Omicron (**Fig. S2c**). Upon first infection with either Delta or Omicron, individuals eventually enter the compartment R_{delta} or $R_{omicron}$, respectively. Individuals can then get infected a second time with Omicron, as the protection conferred by natural immunity in the compartments R_{delta} and $R_{omicron}$ is less than 100%, with different values depending on the strain of the prior infection (summarized in **Table S3**, **Section 3.1** *Infection-induced Immunity*). The final size of the R compartment in the Alpha-Delta model was used as initial condition for the R_{delta} compartment.

We additionally considered strata for vaccination, by splitting all above compartments into subgroups labelled as V0, V1, V2, and V3, indicating unvaccinated or vaccinated individuals with 1, 2, or 3 doses, respectively (**Fig. S2d**). Transitions from V0 to V1, from V1 to V2 and from V2 to V3 are informed from data on the number of vaccine doses administered over time[1]. Vaccine doses in the model are administered not only to S but also to the R compartment, proportionally to their sizes. We considered waning in vaccine effectiveness, modeled as a step-wise decrease in protection after 5 weeks, 10 weeks, and 15 weeks (**Fig. S2e**). Values of initial vaccine effectiveness and waning are summarised in **Table S4** (**Section 3.2** *Vaccine Effectiveness*). In the compartments R_{delta} and $R_{omicron}$, vaccine-induced and infection-induced protection are combined following the rules described in **Section 3.3** *Hybrid Immunity*.

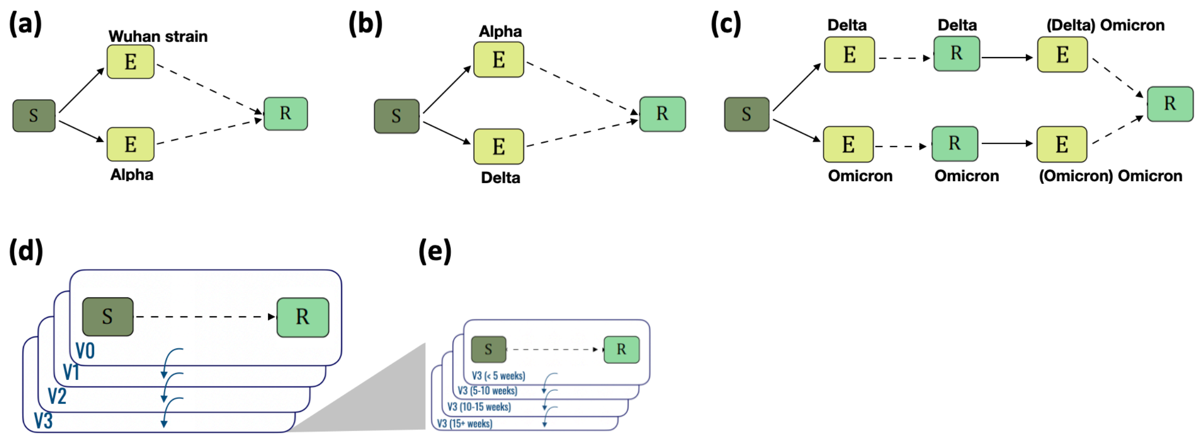

Figure S2. Scheme of additional layers in the transmission model. The dashed arrow indicates disease progression as described in **Fig. S1**. **(a)** Two-strain model considered for the Wuhan and Alpha strain. **(b)** Two-strain model considered for the Alpha and Delta strain. **(c)** Two-strain model considered for the Delta-Omicron epidemic, with waning of immunity and possible re-infection with Omicron**. (d)** Vaccination model accounting for up to 3 doses. Compartments labeled as V0, V1, V2, and V3 indicated unvaccinated or vaccinated with 1, 2, or 3 doses). **(e)** Model with step-wise waning of vaccine-induced immunity.

1.2 Model parameters for the Wuhan strain

Table S1 illustrates the parameters used for the Wuhan strain. See Section 2 for variant-specific parameters.

Table S1. Parameters, values, and sources used to define the compartmental model for the Wuhan strain.

Variable	Description	Value (Wuhan strain)	Source
θ^{-1}	Incubation period	5.2 days	[2]
μ_p^{-1}	Duration of prodromal phase	1.5 days, computed as the fraction of pre-symptomatic transmission events out of pre-symptomatic plus symptomatic transmission events.	[3]
ϵ^{-1}	Latency period	$\theta^{-1} - \mu_p^{-1}$ = 3.7 days	-
p_a	Probability of being asymptomatic	0.4	[4]
p_{ps}	Probability of being pauci-symptomatic	0.4 for children, adolescents<br>0.12 for adults, seniors	[5,6]
p_{ms}	Probability of developing mild symptoms	Computed as 1 – (p_a + p_{ps}+ p_{ss})	-
p_{ss}	Probability of developing severe symptoms	0.002 for children<br>0.001 for adolescents<br>0.011 for adults<br>0.096 for seniors	[7]
g	Generation time	6.6 days	[8]
μ^{-1}	Infectious period for I_a, I_{ps}, I_{ms} , I_{ss}	2.0 days (chosen accordingly to generation time distribution assumed for I_{ms})	-
r_β	Relative infectiousness of I_p, I_a, I_{ps} with respect to I_{ms} and I_{ss}	0.25 for children<br>0.55 for adolescents, adults, seniors	[9]
σ	Relative susceptibility	0.7 for children and adolescents<br>1 for adults, seniors	[6,10–12]
δ^{-1}	Delay from onset to hospitalization	5 days	[13,14]
ω^{-1}	Waiting time for hospitalization	$\delta^{-1} - \mu^{-1}$= 3 days	-
γ^{-1}	Time spent in H	14 days	[13]

2. VARIANT-SPECIFIC PARAMETERS

All variant-specific epidemiological parameters are summarized in **Table S2**. For sources and additional details, see sections 2.1, 2.2, 2.3 and 2.4.

Table S2. Summary table with variant-specific parameters.

**Variant**	**Alpha**	**Delta**	**Omicron**
Generation time	6.6 days	4.7 days	4.2 days
Transmission advantage	+59% wrt Wuhan strain	+100% wrt Alpha	Omicron BA.1: +5% wrt Delta<br>Omicron BA.2: +47 wrt Omicron BA.1
Hospitalization risk	factor 1.64 relative to Wuhan strain	factor 2.28 relative to Wuhan strain	factor relative to Delta:<br>1.0 for children<br>0.78 for adolescents<br>0.2 for adults and seniors
Relative susceptibility of children and adolescents wrt adults and seniors	100% in main analysis<br>70% for sensitivity	100% in main analysis<br>70% for sensitivity	100% in main analysis<br>70% for sensitivity

2.1 Generation time

The generation time for the Alpha variant was assumed to be the same as the Wuhan strain. We modeled a shorter generation time for the Delta and Omicron variant, with mean equal to 4.7 days[15] and 4.2 days[16] respectively. This was achieved by shortening the latent period to 1.8 days and 1.3 days, keeping the total infectious period equal to 3.5 days for each variant.

2.2 Transmission advantage

The variant's transmission advantage is integrated in the model as a multiplicative factor of the transmission rate β for the force of infection due to individuals infected by the variant under consideration.

We used 59% transmission advantage for Alpha with respect to the Wuhan strain, following Ref.[17]. We then used values for transmission advantages estimated using our transmission model and genomic data in our previous work (see **Table S2** of Ref.[18]): 100% transmission advantage of Delta with respect to Alpha, 5% of Omicron BA.1 with respect to Delta and 47% of Omicron BA.2 with respect to Omicron BA.1.

2.3 Hospitalization risk

We used estimates of relative hospitalization risks from Ref.[19] for Alpha and Delta variants with respect to the Wuhan strain, in particular an increased hospital risk by a factor 1.64 for the Alpha variant and 2.28 for the Delta variant.

For Omicron, we used age-specific relative risks with respect to the Delta variant, multiplying the probability of being hospitalized by a factor of 1.0 for children, 0.78 for adolescents, and 0.2 for adults and seniors, in line with estimates for non-vaccinated individuals in Ref[20].

2.4 Susceptibility

We assumed susceptibility of younger individuals (children and adolescents) to be the same as adults when considering the Alpha, Delta and Omicron variant, in absence of studies quantifying the age-dependent susceptibility specific to SARS-CoV-2 variants. For the Wuhan strain, we considered younger individuals to have a lower susceptibility relative to adults, equal to 70%, based on evidence from the literature (**Table S1**). We performed a sensitivity analysis where we assumed the same age-dependent susceptibility for both the Wuhan strain and the variants, i.e. a reduced susceptibility of children and adolescents with respect to adults (**Section 9.2**).

3. POPULATION IMMUNITY

For this section, we assumed the same parameterization used in our previous work (see Supplementary Information in Ref.[18]).

3.1 INFECTION-INDUCED IMMUNITY

We do not model re-infections in the pre-Omicron period, i.e. we assume that infection-induced immunity from variants prior to Omicron fully protects against re-infection for at least 18 months (Ref.[21]). In the Delta-Omicron period, we account for the possibility of experiencing a second infection with Omicron, after a prior Omicron or non-Omicron infection, with infection-induced protection detailed in **Table S3**. These values apply to non-vaccinated individuals. For vaccinated individuals with past infection, we consider hybrid immunity as detailed in **Section 3.3**. We also assumed that, if re-infected, individuals have 50% reduced transmissibility with respect to individuals infected for the first time.

Table S3. Protection against a re-infection and subsequent hospitalizations according to infection history and exposure.

Prior infection	New exposure	Protection against re-infection	Protection against hospitalization	Source
Non-Omicron	Non-Omicron	100%	100%	We neglect re-infections in the pre-Omicron period due to the limited number of cases of re-infections observed up to the Delta wave (Ref.[22,23])
Omicron	Non-Omicron	100%	100%	We neglect Omicron->non-Omicron re-infections due to the fast take-over of the Omicron strain.
Non-Omicron	Omicron	62% within 6 months since prior infection<br>30% after 6 months	88%	[24–26]
Omicron	Omicron	90%	95%	[27]

3.2 VACCINE EFFECTIVENESS

Values of vaccine effectiveness against infection, symptomatic infection, hospitalization and transmission are summarised in **Table S4**. We used available estimates from the literature relative to the Pfizer vaccine, that was the one mainly administered in France[28]. We used the same values of vaccine effectiveness against Omicron BA.1 and BA.2 sub-lineages, as estimates show very limited differences between the two (Ref.[29]). We assumed that protection conferred by vaccination becomes effective 2 weeks after injection in case of 1 or 2 doses[30], and 1 week after injection in case of a third dose[31]. We assumed vaccine effectiveness to be the same for all age groups, in line with Refs.[32,33] showing evidence of similar vaccine effectiveness in adolescents and adults. Values in **Table S4** are in accordance with more recent estimates of rate of waning of vaccine effectiveness from a large systematic review[34].

Table S4. Values for vaccine-induced protection against different outcomes, differentiated by variant. Definitions: VE stands for vaccine effectiveness, w stands for weeks.

VE against	Wuhan and Alpha variant		Delta variant									Omicron variant								
	1 dose	2 doses	1 dose	2 doses 2-5w	2 doses 5-10w	2 doses 10-15w	2 doses >=15w	3 doses 1-5w	3 doses 5-10w	3 doses 10-15w	3 doses >=15w	1 dose	2 doses 2-5w	2 doses 5-10w	2 doses 10-15w	2 doses >=15w	3 doses 1-5w	3 doses 5-10w	3 doses 10-15w	3 doses >=15w
infection	60% [35]	95% [36,37]	60% [+]	81% [37],[*]	77% [*]	71% [*]	63% [*]	86% [*]	81% [*]	81% [*]	81% [*]	24% [^]	52% [^]	38% [^]	24% [^]	11% [^]	52% [^][c]	48% [^][c]	40% [^][c]	28% [^][c]
symptomatic infection	70% [35]	97% [36]	70% [38]	90% [38],[**]	85% [38],[**]	79% [38],[**]	70% [38], [**]	95% [29,38]	90% [29,38]	90% [29,38]	90% [29,38]	30% [38]	65% [**]	47% [**]	30% [**]	14% [**]	65% [29] [c]	60% [29] [c]	50% [29] [c]	35% [29] [c]
hospitalization	80% [35]	97.5% [36]	80% [+++]	97% [39] [***]	97% [39],[***]	97% [39] [***]	97% [39] [***]	98% [39]	98% [39]	98% [39]	98% [39]	60% [-]	74% [40] [***]	68% [40] [***]	62% [40] [***]	55% [40] [***]	85% [39]	85% [39]	75% [39,41]	75%[41]
transmission	15% [42]	68% [42]	15% [42]	50% [42]	50% [42]	24% [42]	24% [42]	50% [++]	50% [++]	24% [++]	24% [++]	15% [--]	50% [--]	50% [--]	24% [--]	24% [--]	50% [--]	50% [--]	24% [--]	24% [--]

[*] VE against infection = 90% of VE against symptoms (from Ref.[43])
[**] quadratic fit of estimates from Ref.[38]
[***] quadratic fit of estimates from Ref.[39]
[+] assumed equal to Alpha variant, and equal to 90% of VE with 2 doses
[++] assumed to be the same as the 2nd dose
[+++] assumed equal to Alpha variant
[^] assumed that VE(infection) = 80% of VE(symptomatic infection)
[c] in line with the consensus table in Ref.[29]
[-] computed assuming same reduction in VE against hospitalization between 2nd and 1st dose for the Delta variant
[--] assumed equal to the Delta variant; in line with following evidence for Omicron (Ref.[44])

3.3 HYBRID IMMUNITY

Let VE, NE, HE be the protection against infection conferred by vaccine only (vaccine effectiveness), prior infection only (natural immunity), and both (hybrid immunity), respectively.

Hybrid immunity in case of past Omicron infection

We combine the levels of protection conferred by vaccine and prior infection by setting HE = max(VE, NE), accounting for waning in both VE and NE.

Hybrid immunity in case of past non-Omicron infection

Some studies[25,45] indicate that the risk of infection in case of vaccination and prior infection is approximately half the risk of infection when vaccinated only, i.e. HE = 1-(1-VE)x0.5. However, we constraint that hybrid immunity must be at least as strong as the natural immunity alone, hence we set HE = max(1-(1-VE)x0.5, NE) for hybrid immunity against infection. For what concerns hospitalization, we assume HE = max(VE, NE), i.e. we consider the higher values between the protection conferred by vaccination only or natural immunity only (accounting for the waning).

The resulting values for natural, vaccine-induced and hybrid immunity against Omicron and Delta are illustrated in Fig. S2 of the Supplementary Information of our previous work (Ref.[18]).

4. PARAMETERIZATION OF CONTACT MATRICES

4.1 SCHOOL CALENDAR

Besides the data presented in the main text in **Fig. 1c,d** (mobility and avoidance of physical contacts) we also used school attendance over time to adjust contacts at school. The timeline of school closure and (partial) opening is reported in **Table S5**.

Table S5. Calendar of school attendance in France. For the exit phase from the first lockdown (11 May – 5 July 2020, period of voluntary attendance), we used data from the Ministry of Education[46] on the observed school attendance.

**Period**	**Attendance in pre-primary and primary schools (children 0-10 y.o.)**	**Attendance in middle and high schools (adolescents 11-18 y.o.)**	**Details**
1 Mar – 16 Mar 2020	100%	100%	Schools regularly open
17 Mar – 10 May 2020	0%	0%	Schools closed (first lockdown)
11 May – 5 July 2020	21%	19%	Exit phase, schools re-opened with voluntary attendance. Data from Ref.[46]
6 July – 30 Aug 2020	0%	0%	Summer holidays
31 Aug – 18 Oct 2020	100%	100%	Schools regularly open
19 Oct – 1 Nov 2020	0%	0%	School holidays (All Saint's break)
2 Nov – 20 Dec 2020	100%	100%	Second lockdown, with school open
21 Dec 2020 – 3 Jan 2021	0%	0%	Christmas holidays
4 Jan – 7 Feb 2021	100%	100%	Curfew period, with schools open
8 Feb – 14 Feb 2021	75%*	75%*	Winter school holidays in zone A
15 Feb – 21 Feb 2021	46%*	46%*	Winter school holidays in zone A and C
22 Feb – 28 Feb 2021	25%*	25%*	Winter school holidays in zone B and C
1 Mar – 7 Mar 2021	54%*	54%*	Winter school holidays in zone B
8 Mar – 4 Apr 2021	100%	100%	Curfew period, with schools open
5 Apr – 11 Apr 2021	0%	0%	School closed (remote learning)
12 Apr – 25 Apr 2021	0%	0%	Spring holidays (national level)
26 Apr – 2 May 2021	100%	0%	Schools open for children; schools closed for adolescents (remote learning)
3 May – 4 July 2021	100%	100%	Schools regularly open
5 July – 29 Aug 2021	0%	0%	Summer holidays
30 Aug – 24 Oct 2021	100%	100%	Schools regularly open
25 Oct – 7 Nov 2021	0%	0%	School holidays (All Saint's break)
8 Nov – 19 Dec 2021	100%	100%	Schools regularly open
20 Dec 2021 – 2 Jan 2022	0%	0%	Christmas holidays
3 Jan – 6 Feb 2022	100%	100%	Schools regularly open
7 Feb – 13 Feb 2022	54%*	54%*	Winter school holidays in zone B
14 Feb – 20 Feb 2022	29%*	29%*	Winter school holidays in zone A and B
21 Feb – 27 Feb 2022	46%*	46%*	Winter school holidays in zone A and C
28 Feb – 6 Mar 2022	71%*	71%*	Winter school holidays in zone C
7 Mar – 10 Apr 2022	100%	100%	Schools regularly open
11 Apr – 17 Apr 2022	54%*	54%*	Spring school holidays in zone B
18 Apr – 24 Apr 2022	29%*	29%*	Spring school holidays in zone A and B
25 Apr – 1 May 2022	46%*	46%*	Spring school holidays in zone A and C
2 May – 8 May 2022	71%*	71%*	Spring school holidays in zone C
9 May – 22 May 2022	100%	100%	Schools regularly open

*Winter and spring school holidays in France are set at a sub-national level, alternating departments grouped in 3 zones (zone A, B and C)[47]. Values in the table refer to the effective school attendance at national level, computed as a weighted average of the population of students in zones with school holidays (0% attendance) and zones with schools in session (100% attendance).

4.2 CONSTRUCTION OF SYNTHETIC MATRICES

Here we describe the framework we used to build the weekly mobility-based synthetic matrices.

First, let M be the pre-pandemic baseline matrix, whose entries M_{ij} represent the average daily number of contacts that an individual in participant age group i establishes with individuals in the contact age group j. This matrix was corrected for the French demography in 2020, as described in the main text (Methods). The matrix M is reciprocal, i.e. the total number of contacts declared by individuals in age group i with j is equal to the number declared by individuals in group j with group i, i.e. $M_{ij}N_i = M_{ji}N_j$ with N_i, N_j corresponding to the age profile of the French population in the year 2020. For discussions about demography and reciprocity correction, see for example Ref.[48].

Then, we compute the contact matrix C as

$$C_{ij} = \frac{M_{ij}}{N_j} N$$

with N the total French population in 2020. Given that the matrix M is reciprocal, the resulting matrix C will be symmetric. We will call this matrix $C_{baseline}$, where the element C_{ij} is the *per capita* contact rate between age group i and j (rescaled by a global factor N). The definition of the matrix C is equivalent to the "intrinsic connectivity matrix" introduced in Ref.[48] . In other words, C_{ij} is the expected number of contacts formed by a group-i individual with group-j individuals, if 100% of the population was in group- j, or equivalently, C_{ij}/N is the expected number of contacts formed by a group-i individual with group-j individuals per individual in group-j.

Contacts are reported by location and by type of contact. Therefore, the matrix C_{baseline} will be made of the following layers:

$$C_{baseline} = C_{home} + C_{work} + C_{school} + C_{transport} + C_{leisure} + C_{other}$$

and for each location ($location \in \{home, work, school, transport, leisure, other\}$):

$$C_{location} = C_{location}^{non-physical} + C_{location}^{physical}$$

Finally, we construct synthetic time-varying contact matrices $C(t)$ by reducing contact rates in the different layers of the baseline matrix $C_{baseline}$. We will apply symmetric reductions (i.e. both on rows and columns) to preserve the symmetry of the matrix C and therefore the reciprocity of the matrix M. Synthetic matrices are constructed on a weekly basis (except for lockdown matrices which were defined for the period of implementation of the measure) and integrated in the transmission model with a daily time step.

In the following sections 4.2.1 – 4.2.5, we describe in detail the reductions applied to the different layers. In section 4.2.6, we provide a summary table as an overview of the full framework (**Table S6**).

4.2.1 MODELING TELEWORK

Working from home was implemented as a social distancing strategy throughout the pandemic in France. We assume that telework has an impact on reducing contacts at work and on transports.

Reducing contacts at work

We apply the following to reduce contacts at work:

$$\hat{C}_{work} = C_{work} * \frac{((1 - a_{TW}) + (1 - a_{TW})^2)}{2}$$

where a_{TW} is the percentage of workers performing telework, informed weekly as the reduction in mobility related to workplaces, from Google mobility data. More precisely, Google measures the change in the number of visitors to a specific location with respect to a pre-pandemic baseline (February 2020); therefore, the

mobility change related to workplaces can be interpreted as the reduction in attendance at work, or in other words as the fraction of people performing telework.

The formula above was laid down using the following reasoning. We aimed at reducing contacts at work based on the reduced presence in workplaces due to telework. The matrix element $C_{work}(i,j)$ is the per-capita contact rate between age group i and age group j in a work setting. Let p_i and p_j be the fraction of workers of age i and j respectively who do not go to work. Then, we reduce the per-capita contact rate in the workplace to $C_{work}(i,j) * (1-p_i) * (1-p_j)$, accounting for the reduction in the number of individuals of age i (age of person making the contact) and j (age of contacted person) present in the workplace. This can be also interpreted as a *density-dependent* assumption on the relation between contact rates and population density[49]. In other words, we are performing a weighted average where p_i individuals who are not going to work have 0 contacts, while the rest $(1-p_i)$ will have a reduced number contacts $C_{work}(i,j)*(1-p_j)$ scaled by the reduction in the density of available contactees. However, contacts at work are declared as such by the participant in the contact survey, but may in principle be engaged with individuals who are not working (e.g. a bus driver, a customer-facing employee, etc.). In that case, we would reduce the contact rate as $C_{work}(i,j) * (1-p_i)$. This can also be interpreted as a *frequency-dependent* assumption on the relation between contact rates and population density[49]. In other words, we are performing a weighted average where p_i individuals who are not going to work have 0 contacts, while the rest $(1-p_i)$ will have the same number contacts $C_{work}(i,j)$, regardless of the density of available contactees in the workplace. In absence of data to inform the frequency of the two situations, we used the average between the two. Also, we take $p_i = p_j = a_{TW}$ regardless of age. Finally, we obtain the equation described above.

Reducing contacts on transports

We apply the following to reduce contacts on transports:

$$\hat{C}_{transport} = C_{transport} \odot A_{transport}$$

where $\odot$ is the element-wise product, $A_{transport}(i,j) = (1-p_i) * (1-p_j)$ and p_i, p_j is the fraction of individuals who do not go on transportations due to telework or remote schooling in age group i and j respectively.

For the age groups of *adults and seniors*, we set $p_i = w_i * a_{TW} + (1-w_i) * x_{TR}$ where

- w_i is the proportion of workers in the age-group i (i.e. the active population), set at 78% and 3% for adults and seniors respectively, informed from labor force statistics[50];
- a_{TW} is the percentage of workers performing telework, informed from mobility data as described above;
- x_{TR} is the fraction of non-workers individuals on transports who do not take transports anymore due to the ongoing epidemic; we used x_{TR} = 75% as an assumption throughout the whole pandemic, starting from the first lockdown; contacts on transports do not constitute a large fraction (see **Fig. 1b** in the main text), so this assumption is expected to have a small impact on the synthetic matrix.

Similarly to the work matrix, given a fraction p_i of individuals who do not go on transportation, those individuals will have 0 contacts on transports; we assume that the remaining $1-p_i$ has a p_j reduction in contacts $C_{ij}^{transport}$; we take a weighted average obtaining $(1-p_i) * (1-p_j) * C_{ij}^{transport}$. In other words, we are assuming density-dependent contact activity on transports, which is reasonable in this context.

For the age groups of *children and adolescent,* we assume that they take transports mainly to go to school, so p_i is the fraction of young people not attending school (see sections below for additional details).

4.2.2 Modeling partial or full school closure

In this section, we refer to school closure as closure during a lockdown, i.e. with distance learning (remote school). We model school holidays (with students not going to school but likely performing other activities rather than staying at home) in a different way (see section *School holidays* below).

We assume that school closure (distance learning) has an impact on reducing contacts at school and reducing contacts on transports.

Reducing contacts at school

$\hat{C}_{school} = C_{school} \odot A_{school}$ where $A_{school}(i,j) = (1 - p_i) * (1 - p_j)$ and

- for i = children, adolescents: p_i is the fraction of students not attending school; so $p_i = 1$ is schools are fully closed; $p_i = 0$ if schools are fully opened; $p_i \in (0,1)$ if they are opened with limited attendance, e.g. in the exit phase from the first lockdown when schools reopened with voluntary attendance (see **Table S5**);
- for i = adults, seniors: p_i is the fraction of school personnel not going to school; so p_i = 1 if schools are fully closed; p_i = 0 if schools are partially opened of fully opened; we assumed that even if attendance of students is partial, teachers and administrative staff are fully present.

Reducing contacts on transports

$\hat{C}_{transport} = C_{transport} \odot A_{transport}$, where $\odot$ is the element-wise product, and $A_{transport}(i,j) = (1 - p_i) * (1 - p_j)$ where p_i is the fraction of students not going to school; for adults, seniors, p_i was defined above based on telework (see section *Modeling telework*).

4.2.3 Leisure and other activities

During the first and second lockdown periods, we completely removed contacts associated with leisure and other activities, setting $\hat{C}_{leisure} = 0$, $\hat{C}_{other} = 0$. Outside of the first and second lockdown, we assumed a 50% reduction (i.e. $\hat{C}_{leisure} = 0.5 * C_{leisure}$ and analogously for *other*) for the first year and half of the pandemic, including the third lockdown period which was milder in terms of stringency of interventions. Starting from May 3, 2021 (week 18, after the end of the third lockdown), we assumed full contacts in leisure and other activities.

4.2.4 Avoidance of physical contacts

For any location except *home*, before applying the reductions based on the rules set above, we first reduced the number of physical contacts, e.g. for the work matrix

$$\hat{C}_{work} = (C_{work}^{non-physical} + (1-q) * C_{work}^{physical}) * \frac{((1 - a_{TW}) + (1 - a_{TW})^2)}{2}$$

where q is the fraction of population declaring avoidance of physical contacts in the Santé publique France CoviPrev survey[51].

We assumed the reduction of physical contacts to be the same across the age classes. It is also reasonable to think that students at school may have avoided physical contacts by replacing non-physical contacts. In other words, the reduction of physical contacts at school (or other setting) for young individuals (children and adolescents) may have been too strong in the construction of the synthetic contact matrices. This has been discussed as a limitation in the main text.

4.2.5 School holidays

For summer holidays, we used the equations set for modeling the effect of partial school closure (hence reducing contacts in the school and transports layers of the matrix, see section *Modeling partial or full school closure*) assuming 80% reduction in attendance. The idea behind is to keep a fraction of school-related contacts to account for summer activities such as school camps involving students' interactions.

For autumn, Christmas, winter and spring holidays, we used the spring holiday school matrix, and the spring holiday transport matrix. The original pre-pandemic survey collected data for a regular weekday (that we used throughout the paper) and for holidays (in this case, spring school holidays). We used the latter source of contacts to model the effect of school holidays, in particular we used the school matrix layer, and the

transport matrix (for the latter, we use only the matrix elements involving children and adolescents; for the other elements of the matrix, we used the contacts reported for a weekday, adjusted for telework as described in the previous sections).

4.2.6 Summary table

In **Table S6** we summarize the framework used to build the weekly contact matrices.

Table S6. Details on the construction of the weekly contact matrices.

**Period**	**Week number**	**Telework (individuals not going to work)**	**Schools**	**Leisure and other non-essential activities activities**	**Physical contacts (outside home)**
First lockdown (LD1)	w12-19, 2020	63% (Orange mobile phone data[52], used in absence of Google mobility data)	Schools closed (distance learning, 100% contact reduction in school and on transports)	100% reduction in contacts	Weekly reduction based on survey data
Exit phase	w20-27, 2020	Weekly mobility reduction (Google data)	Partial attendance (ministry of education data)	50% reduction in contacts	Weekly reduction based on survey data
Summer 2020	w28-35, 2020	Weekly mobility reduction (Google data)	Summer holidays (modeled with partial attendance 20%)	20% contact reduction among children and adolescents; 50% reduction otherwise	Weekly reduction based on survey data
Autumn 2020	w36-42, 2020	Weekly mobility reduction (Google data)	Full attendance	50% reduction	Weekly reduction based on survey data
Autumn school holidays	w43-44, 2020	Weekly mobility reduction (Google data)	Spring holiday contact matrix for school (M_{ij} for any age group i, j) and transport (only M_{ij} with age group $i, j \in \{children, adolescents\}$)	50% reduction	Weekly reduction based on survey data
Second lockdown (LD2), full adherence	w45-47, 2020	Mobility reduction (Google data), averaged over the three weeks	Full attendance	100% reduction	Weekly reduction based on survey data
Second lockdown (LD2), loss of adherence / gradual reopening	w48-51, 2020	Weekly mobility reduction (Google data)	Full attendance	100% reduction	Weekly reduction based on survey data
Christmas	w52-53, 2020	Weekly mobility reduction (Google data)	Spring holiday contact matrix for school (M_{ij} for any age group i, j) and transport (only M_{ij} with age group $i, j \in \{children, adolescents\}$)	50% reduction	Weekly reduction based on survey data
Curfew with school in sessions	w1-5, 2021<br><br>w10-13, 2021	Weekly mobility reduction (Google data)	Full attendance	50% reduction	Weekly reduction based on survey data
Winter school holidays	w6-9, 2021	Weekly mobility reduction (Google data)	Weighted average of school attendance based on zone A,B,C	50% reduction	Weekly reduction based on survey data
Third lockdown (LD3), school closed	w14, 2021	Weekly mobility reduction (Google data)	Schools closed (100% contact reduction in school and on transports)	50% reduction	Weekly reduction based on survey data
Third lockdown (LD3), Spring school holidays	w15-16, 2021	Weekly mobility reduction (Google data)	Spring holiday contact matrix for school (M_{ij} for any age group i, j) and transport (only M_{ij} with age group $i, j \in \{children, adolescents\}$)	50% reduction	Weekly reduction based on survey data
Third lockdown (LD3), partial school reopening	w17, 2021	Weekly mobility reduction (Google data)	School open for children, distance learning for adolescents	50% reduction	Weekly reduction based on survey data
Exit phase with curfew	w18-26, 2021	Weekly mobility reduction (Google data)	Full attendance	No reduction	Weekly reduction based on survey data
Summer 2021	w27-34, 2021	Weekly mobility reduction (Google data)	Summer holidays (modeled with partial attendance 20%)	No reduction	Weekly reduction based on survey data
From Autumn 2021 onwards, we apply the same rules as of Autumn 2020 with regular school holidays and full contacts in leisure and other activities.					

4.3 Adaptation and integration of the SocialCov contact matrices

The empirical contact matrices, estimated from the SocialCov survey data, were computed for the following age classes: [0-10], [11-17], [18-19], [20-29], [30-39], [40-49], [50-59], [60-64], [65-69], 70+. We needed to align these age groups to the ones used of the synthetic contact matrices, i.e. [0-10], [11-18], [19-64], 65+. To do so, we applied the following procedure.

We first split the age group i =[18-19] into $i_1 = 18$ and i_2 = 19, assuming the same contact rate $M_{ij} = M_{i_1 j} = M_{i_2 j}$ for $j \neq$ [18-19], and distributing the number of contacts M_{ii} within group i proportionally to the population in age groups i_1 and i_2, i.e. $M_{i_1,i_1} = M_{ii} * (N_{i_1}/N_i)$ and $M_{i_1,i_2} = M_{ii} * (N_{i_2}/N_i)$, and analogously $M_{i_2,i_1} = M_{ii} * (N_{i_1}/N_i)$ and $M_{i_2,i_2} = M_{ii} * (N_{i_2}/N_i)$. We then aggregated over the four age groups of interests, summing over the contact age groups j and performing a weighted average over the age groups i of the participants.

The social contact survey carried out during the first lockdown did not collect data for young individuals but only for adults. We imputed the missing number of contacts for individuals in [0-10] and [11-18] using contacts of the pre-pandemic home matrix. This was a reasonable assumption as the other elements of the matrix were highly similar to the pre-pandemic home matrix.

Finally, we had seven empirical contact matrices, estimated with contacts collected in the periods displayed in **Table S7**.

Table S7. Periods of data collection of the SocialCov surveys.

Survey period	Matrix name	NPIs	Schools	Corresponding synthetic matrix
Apr 10-28, 2020	LD1	First lockdown	Closed	Matrix for the first lockdown (w12-19, 2020)
Dec 9-22, 2020	M1	Curfew 8pm	Open	Matrix for w51, 2020 (Dec 14-20)
Jan 10-21, 2021	M2	Curfew 6pm	Open	Matrix for w2, 2021 (Jan 11-17)
Mar 2-10, 2021	M3	Curfew 6pm	Open	Matrix for w9, 2021 (Mar 1-7)
Aug 12-24, 2021	M4	Summer	Closed	Matrix for w33, 2021 (Aug 16-22)
Dec 6-17, 2021	M5	Health pass	Open	Matrix for w49, 2021 (Dec 6-12)
May 20-29, 2022	M6	- - -	Open	Matrix for w20, 2022 (May 15-21)

These empirical contact matrices were then extended over time to be integrated in the transmission model, as pictured in **Fig. 5b**. We used matrix M4 (estimated for summer 2021) as a proxy for school holidays, as this was the survey wave occurring in a period with schools closed (excluding the lockdown). We extended the periods of M1, M2, M3, M5 and M6 as appropriate, intercut by the use of matrix M4.

4.4 Modeling the impact of testing and self-isolation

After computing the matrices with the two approaches (mobility-based synthetic matrices and survey-based empirical matrices), we integrated them in the transmission model presented in **Fig. S1**.

We modeled a spontaneous change of behavior due to severe illness. We assumed that infectious individuals with severe symptoms self-isolate and spontaneously reduce by 75% their number of contacts because of the illness they experience, as observed during 2009 H1N1 pandemic[53]. Therefore, the contact matrix associated with the compartment I_{ss} in the force of infection of the transmission model is multiplied by a factor 0.25. This factor is not applied to the other non-severe infectious stages (I_p, I_a, I_{ps}, I_{ms}).

To simulate the effect of a test-trace-isolate strategy, we considered that a given fraction of infectious individuals reduces their contacts by 90% throughout their illness as they are put into isolation after a positive test. We modeled this effect implicitly by using a weighted contact matrix, without adding an explicit compartment for the tested individuals. Let p_t be the fraction of infectious individuals identified as positive, and C the contact matrix; then in the force of infection we used $C(x) = 0.1 * p_t * C + (1 - p_t) * C$ for the compartments $x = I_a, I_{ps}, I_{ms}$. For individuals with severe symptoms I_{ss}, we combined this additional

reduction with the change of behavior explained above, by using $C(I_{ss}) = 0.1 * p_t * C + 0.25 * (1 - p_t) * C$. We assumed $p_t = 0$ during the first wave (pre-lockdown and first lockdown period), as no systematic testing system was in place at that time in France. We assumed $p_t = 50\%$ from the exit phase (May 2020) onwards. To account for a delay in tracing, testing, and self-isolation, we considered that infected individuals in their prodromic stage I_p maintain their contacts, without applying any reduction, as done in previous works[18,54,55]. We note that the choice adopted to model the effect of isolation assumes a density-dependent effect, as the contacts removed due to isolation are not replaced with other contacts among non-isolated individuals. An alternative frequency-dependent approach would require additional complexity, but it is however expected to have a negligible effect as prevalence in the I_{ss} compartment is limited in size.

5. METRICS USED TO COMPARE CONTACT MATRICES

In this section, we describe the metrics that we used in the main text to compare the contact patterns obtained with the two approaches, i.e. the synthetic matrices and the survey-based matrices.

5.1 Average connectivity

Let M be the contact matrix, where the element M_{ij} represents the average number of contacts that an individual in age group i establishes with individuals in age group j.

We can compute the overall average number of contacts as $< k > = \sum_{i,j} N_i M_{ij}/N$ and the total number of contacts for age group i as $k_i = \sum_j M_{ij}$.

5.2 Cosine similarity

In **Fig. S10a**, we used the cosine similarity as a measure of similarity between the synthetic contact matrices and the empirical contact matrices, as done in Ref.[56]. To compute the cosine similarity, each matrix is first transformed into a vector by concatenating its rows. The cosine similarity between two vectors v and w is defined as $(\sum_h v_h w_h)/(\sum_h v_h^2 * \sum_h w_h^2)$.

5.3 Proportion of young connectivity

For this metric, we borrowed the definition of proportion of young connectivity introduced in Ref.[48], that is

$$Y = \sum_{i<19,j} \frac{N_i M_{ij}}{\sum_{h,k} N_h M_{hk}} = \sum_{i<19,j} \frac{\frac{N_i M_{ij}}{N}}{< k >}$$

where $< k >$ is the average connectivity as defined above. The quantity Y can be interpreted as the contribution made by young individuals (age groups $i \in \{[0-10],[11-18]\}$) to the overall connectivity. It can also be interpreted as the probability that the contact is produced by a young individual, given the observation of a (directed) contact pair (see Ref.[57]). Let $f_c(i,j)$ be the probability density of a contact pair (i,j) between age group i and age group j. With the contact matrix notation, this is equal to

$$f_c(i,j) = \frac{\frac{N_i}{N} * \frac{M_{ij}}{N_j} * \frac{N_j}{N}}{\sum_{h,k} \frac{N_h}{N} * \frac{M_{hk}}{N_k} * \frac{N_k}{N}} = \frac{N_i M_{ij}}{\sum_{h,k} N_h M_{hk}}$$

hence the definition of Y corresponds to the probability of a contact pair (i,j) with $i < 19$ y.o., using the above probability density.

5.4 Assortativity index

In **Fig. S10b**, we use a definition of age-assortativity index A inspired by Ref.[57].

Assortativity in contact patterns emerges when individuals have a preference of engaging contacts with individuals with similar characteristics. In our case, the characteristic of interest is age. Following the notation of Ref.[57], let $b(s,t)$ be the (per-capita) contact rate, i.e. a bivariate function describing the per-capita rate (per unit time) at which an individual of age $S = s$ makes a contact with an individual of age $T = t$. Let $f(s)$ denote the density of the population age distribution. Then the contact pairs within the population have density

$$f_c(s,t) = \frac{f(s)b(s,t)f(t)}{\int\int f(u)\, b(u,v)\, f(v) du\, dv}$$

With the discrete notation of social contact matrices, that is equal to

$$f_c(i,j) = \frac{\frac{N_i}{N} * \frac{M_{ij}}{N_j} * \frac{N_j}{N}}{\sum_{h,k} \frac{N_h}{N} * \frac{M_{hk}}{N_k} * \frac{N_k}{N}} = \frac{N_i M_{ij}}{\sum_{h,k} N_h M_{hk}}$$

Then, a natural index of absolute disassortativeness is the mean-squared deviation from perfect assortativity (i.e., situation where each individual has contacts only with individuals in the same group)

$$I^2 = \frac{1}{2} E_c[(S-T)^2] = \frac{1}{2}\int\int (s-t)^2 f_c(s,t)\ ds\, dt$$

where E_c is the expectation with respect to the probability density f_c. In social contact matrix terms, that is equivalent to

$$I^2 = \frac{1}{2}\sum_{i,j}(i-j)^2\ \frac{N_i M_{ij}}{\sum_{h,k} N_h M_{hk}}$$

The index I^2 takes values in $[0, +\infty)$. It is equal to 0 if and only if the contact pair (s,t) with $s \neq t$ occurs with probability zero, that is, when contacts are completely assortative, and individuals are in contact only with individuals in their same group. Increasingly disassortative contacts result in increasingly large positive values of I^2.
A special value of I^2 of particular interest is the one corresponding to homogeneous mixing, i.e. when $b(s,t) = b$ for any s,t. In that case, the degree of disassortativeness is determined entirely by the population density $f(s)$, because we have

$$I^2 = \frac{1}{2}\int\int (s-t)^2 \frac{f(s)\, b\, f(t)}{\int\int f(u)\, b\, f(v) du\, dv}\ ds\, dt = \int\int (s-t)^2 f(s)f(t)\ ds\, dt = \sigma^2$$

with σ^2 the variance of the age distribution. Therefore, a standardized measure of disassortativeness can be defined as $I_s^2 = \frac{I^2}{\sigma^2}$.

We computed this quantity for each contact matrix, using the age distribution of the French population over the four age classes of consideration. In **Fig. S10b**, we plot the assortativity index A defined as

$$A = 1 - I_s^2$$

This index will vary between 0 (homogeneous mixing) and 1 (perfect assortative mixing).

5.5 Proportion of within-group contacts

We also computed the fraction of within-group contacts out of total contacts for each age group, i.e. $\mathrm{M_{ii}}/\sum_j \mathrm{M_{ij}}$ for each age group i. This can be interpreted as a measure of age-specific assortativity. We computed this measure for synthetic and empirical matrices, and also in the pre-pandemic matrix as a reference (**Fig. 4c,d**). This measure provides an indication about the preference of a given age-group in engaging assortative contacts (i.e. contacts with the same age group). In a random mixing scenario, the quantity $\mathrm{M_{ii}}/\sum_j \mathrm{M_{ij}}$ would correspond to the population size $\mathrm{N_i}/\sum_j \mathrm{N_j}$.

6. INFERENCE FRAMEWORK

The force of infection operating on the compartment S_i (susceptible individuals in age class i) at time t is of the general form

$$\lambda_i(t) = \beta(t) \sum_j C_{i,j}(t) * I_j(t)/N$$

where β is the transmission rate per contact, I_j is the number of infectious individuals of age class j, N is the total population and $C_{i,j}$ is the contact matrix defined as $C_{ij} = \frac{M_{ij}}{N_j} N$ (see **Section 4.2**). Note that, given the definition of C_{ij}, the force of infection written above is equivalent to the usual form $\lambda_i(t) = \beta(t) \sum_j M_{i,j}(t) * I_j(t)/N_j$ where the denominator N_j is the number of individuals with age j. This force of infection is then adapted in the model accounting for the relative infectiousness depending on age, disease stage and symptom severity (prodromic, asymptomatic or symptomatic), change of behavior due to severe illness or testing and self-isolation, age-dependent susceptibility, transmission advantage of the variant, and vaccine effectiveness in reducing transmission and susceptibility to infection. For a full derivation of the force of infection, see Chapter 2 of Ref.[58]. Here below, we report the essential steps.

The force of infection is first extended to account for age-dependent susceptibility and infectiousness, and contacts dependent on disease stage, with the following form:

$$\lambda_i(t) = \sigma_i \, \beta(t) \sum_x \sum_j r_\beta(x, j) * C_{i,j}(x, t) * I_{x,j}(t)/N$$

where the index x indicates the different infectious compartments, i.e. $I_p, I_a, I_{ps}, I_{ms}, I_{ss}$, and the indexes i, j indicate the age group. The parameter $\sigma_i \in [0, 1]$ indicates the age-dependent relative susceptibility ($\sigma_i = 1$ for adults and seniors, see **Table S1** for the Wuhan strain). The parameter $r_\beta \in [0, 1]$ is used to modulate the relative infectiousness according to both age and degree of symptoms ($r_\beta = 1$ for adults and seniors in the compartments I_{ms}, I_{ss}, see **Table S1**). The contact matrix C depends both on time (calendar of matrices detailed in **Fig. 5b**) and on the infectious stage x, as detailed above in **Section 4.4**.

Then, we extend the force of infection to account for two-strain dynamics with two variants $s \in \{Wuhan, Alpha\}$, or $s \in \{Alpha, Delta\}$ or $s \in \{Delta, Omicron\}$. The force of infection becomes

$$\lambda_i(t) = \sum_s \sum_x \sum_j \sigma_{i,s} * r_\beta(x, j) * \beta_s(t) * C_{i,j}(x, t) * I^s_{x,j}(t)/N$$

where we sum two contributions depending on the strain s. For each strain s, we take the number of individuals $I^s_{x,j}(t)$ infected with strain s, and the variant-specific transmission rate $\beta_s(t)$ which is defined in terms of transmission advantage with respect to previous strains, e.g. $\beta_{Alpha} = 1.56 * \beta_{Wuhan}$. The parameter $\sigma_{i,s}$ now refers to the age-dependent susceptibility specific for variant s. The values for the transmission advantage and susceptibility for each variant were detailed in **Table S2**.

Finally, we extend the force of infection to account for vaccination status. We write down the force of infection operating on the susceptible individuals in age group i with vaccination status w

$$\lambda^w_i(t) = \sum_v \sum_s \sum_x \sum_j \sigma_{i,s} * \left(1 - VE_{inf}(s, w)\right) * r_\beta(x, j) * \beta_s(t) * \left(1 - VE_{transm}(s, v)\right) * C_{i,j}(x, t) * I^{s,v}_{x,j}(t)/N$$

Here, vaccination status v, w refer to a given number of doses and a given stage of waning in effectiveness. This extension of the force of infection sums the contribution from infected individuals with any vaccination status v, accounting for vaccine effectiveness against transmission $VE_{transm}(s, v)$ which depends both on vaccination status v and the virus strain s. The parameter $\sigma_{i,s}$ is modulated with a factor $\left(1 - VE_{inf}(s, w)\right)$ which implements the protection against infection with virus strain s conferred by the vaccination status w of the susceptible compartment under consideration. The values for $VE_{transm}(s, v)$ and $VE_{inf}(s, w)$ were detailed in **Table S4**. In the Delta-Omicron model, which allows for possible re-infection, we write a similar force of infection for the compartments R_{delta} and $R_{omicron}$.

The transmission rate per contact $\beta(t)$ is inferred as described below. We fitted the model to age-stratified daily hospital admission data and age-stratified seroprevalence data, using a maximum likelihood approach. We used daily hospital data extracted from official national statistics (SIVIC database[60]), corrected for notification delays. We used seroprevalence data, corrected for sensitivity and specificity of the serological test, estimated from six nationwide serological studies (March, April, May, October 2020; February, June 2021). We fitted the transmission rate per contact sequentially, in successive time windows. Each time window represents a pandemic phase, defined by the interventions implemented (e.g., lockdown, curfew, and other restrictions) and activity of the population (school holidays, summer holidays, etc.). More specifically, prior to the first lockdown and in absence of intervention (January–March 2020), we estimated $\{\beta_{pre-LD}, t_0\}$ where β_{pre-LD} is the transmission rate per contact (for the Wuhan strain) and t_0 is the date of the start of the simulation. Then, in each phase we estimated α_{phase}, i.e. a correcting factor of the transmission rate per contact specific to the pandemic phase under consideration (e.g., first lockdown, exit from lockdown, summer, start of second wave, second lockdown, etc.). Practically, this means that the transmission rate $\beta(t)$ is time dependent and it is equal to $\beta_{pre-LD} * \alpha_{phase}(t)$. The time windows for the different phases are listed in **Table S8**; they have a length of 3 weeks on average. The correcting factor α_{phase} absorbs changes over time in the transmission rate that are not modeled through the parameterization of the contact matrices, such as seasonality, use of masks, changes in contact duration; it does not include the effect of vaccination on reducing transmission or the transmission advantage of the variant, which are modeled explicitly through other parameters (**Section 2.2** and **Section 3.2**).

The likelihood function $L(Data|\Theta)$ is of the form

$$\prod_{t=t_1}^{t_n}\prod_{age} P_{Poiss}\left(H_{obs}^{age}(t)\,\middle|\,H_{pred}^{age}(t,\Theta)\right)\cdot P_{Binom}\left(n_{pos}^{age}(t)\,\middle|\,n_{tests}^{age}(t),\pi_{pred}^{age}(t,\Theta)\right)$$

where Θ indicates the set of parameters to be estimated, and $[t_1, t_n]$ is the time window considered for the fit. Additionally, $H_{obs}(t)$ is the observed number of hospital admissions on day t, $H_{pred}(t,\Theta)$ is the median number of hospital admissions generated by the model on day t using parameter values Θ, $P_{Poiss}\left(\cdot\,\middle|H_{pred}(t,\Theta)\right)$ is the density of a Poisson distribution with mean $H_{pred}(t,\Theta)$. Finally, $P_{Binom}(\cdot \mid n,p)$ is the density of a Binomial distribution with probability $p=\pi_{pred}^{age}(t,\Theta)$ being the seroprevalence estimated by the model, and $n=n_{tests}^{age}(t)$ is the number of tests performed in the serological study; lastly, $n_{pos}^{age}(t)$ is the number of seropositive individuals (after correction for sensitivity and specificity of the test).

The parameter space was explored through grid-search and the log-likelihood function was computed to identify the maximum likelihood estimator (MLE). We fitted the model in successive time windows covering a two-year pandemic trajectory from March 1, 2020 to May 22, 2022. The correcting factor alpha is calibrated sequentially in each phase, building on the results of the previous phases.

The baseline transmission rate per contact β_{pre-LD} was estimated at 0.0860 [95% CI 0.0855-0.0865]. The fitted correcting factor is displayed in **Fig. 6a,b** in the main text.

Table S8. Time windows used to fit the correcting factor.

Time window (weeks)	Epidemic phase	Model
w10 – w11, 2020	Rise of 1st wave	Single-strain
w12 – w19, 2020	First lockdown*	Single-strain
w20 – w27, 2020	Exit phase	Single-strain
w28 – w35, 2020	Summer	Single-strain
w36, 2020	School reopening	Single-strain
w37 – w40, 2020	Epidemic slowdown	Single-strain
w41 – w44, 2020	Rise of 2nd wave, with school holidays	Single-strain
w45 – w47, 2020	Second lockdown*	Single-strain
w48 – w51, 2020	Exit phase	Single-strain
w52, 2020 – w2, 2021	Christmas holidays and school reopening**	Two-strain (Wuhan-Alpha)
w3 – w4, 2021	Curfew 6pm	Two-strain (Wuhan-Alpha)
w5 – w6, 2021	Curfew 6pm, start of school holidays	Two-strain (Wuhan-Alpha)
w7 – w9, 2021	School holidays	Two-strain (Wuhan-Alpha)
w10 – w11, 2021	Rise of 3rd wave	Two-strain (Wuhan-Alpha)
w12 – w13, 2021	Third lockdown, schools open	Two-strain (Wuhan-Alpha)
w14 – w17, 2021	Third lockdown, school holidays	Two-strain (Wuhan-Alpha)
w18 – w22, 2021	Exit phase	Two-strain (Wuhan-Alpha)
w23 – w26, 2021	Exit phase, emergence of Delta variant	Two-strain (Alpha-Delta)
w27 – w30, 2021	Rise of 4th wave	Two-strain (Alpha-Delta)
w31 – w34, 2021	Decline of 4th wave	Two-strain (Alpha-Delta)
w35 – w39, 2021	School reopening	Two-strain (Alpha-Delta)
w40 – w42, 2021	Pre-holidays	Two-strain (Delta-Omicron)
w43 – w44, 2021	School holidays	Two-strain (Delta-Omicron)
w45 – w47, 2021	Post-holidays	Two-strain (Delta-Omicron)
w48 – w50, 2021	Epidemic slowdown	Two-strain (Delta-Omicron)
w51 – w52, 2021	Christmas holidays	Two-strain (Delta-Omicron)
w1 – w3, 2022	School reopening, rise of 5th wave	Two-strain (Delta-Omicron)
w4 – w5, 2022	Decline of 5th wave	Two-strain (Delta-Omicron)
w6 – w9, 2022	School holidays	Two-strain (Delta-Omicron)
w10 – w11, 2022	Emergence of Omicron BA.2	Two-strain (Delta-Omicron)
w12 – w14, 2022	Pre-holidays, rise of 6th wave	Two-strain (Delta-Omicron)
w15 – w18, 2022	School holidays, decline of 6th wave	Two-strain (Delta-Omicron)
w19 – w20, 2022	Post-holidays	Two-strain (Delta-Omicron)

*The starting date of the time window considered for the fit of the first and second lockdown is not fixed a priori at the start of the week (Monday), but it is jointly fitted with the correcting factor, in order to account for the actual day of implementation of the lockdown (not on Monday) and also for the delay between the implementation of the measure and the visible effect on hospitalizations (see also the supplementary material in our previous work, Ref.[55]). This allowed the model(s) to better capture the peak observed in the hospital data.
**The time window w52, 2020 – w2, 2021 was used for fitting the model informed with mobility-driven synthetic matrices, and the model using a static pre-pandemic matrix. When fitting the model using the survey-based contact matrix, the time window was split into two, i.e. w52-53, 2020 and w1-w2, 2021, as the sudden change from matrix M4 to matrix M2 did not allow to fit a single correcting factor for the whole period.

7. TRANSMISSION MODEL COMPARISON

7.1 MODEL FIT

In **Fig. S3**, we show the model uncertainty around the median trajectories shown in **Fig. 5a** in the main text.

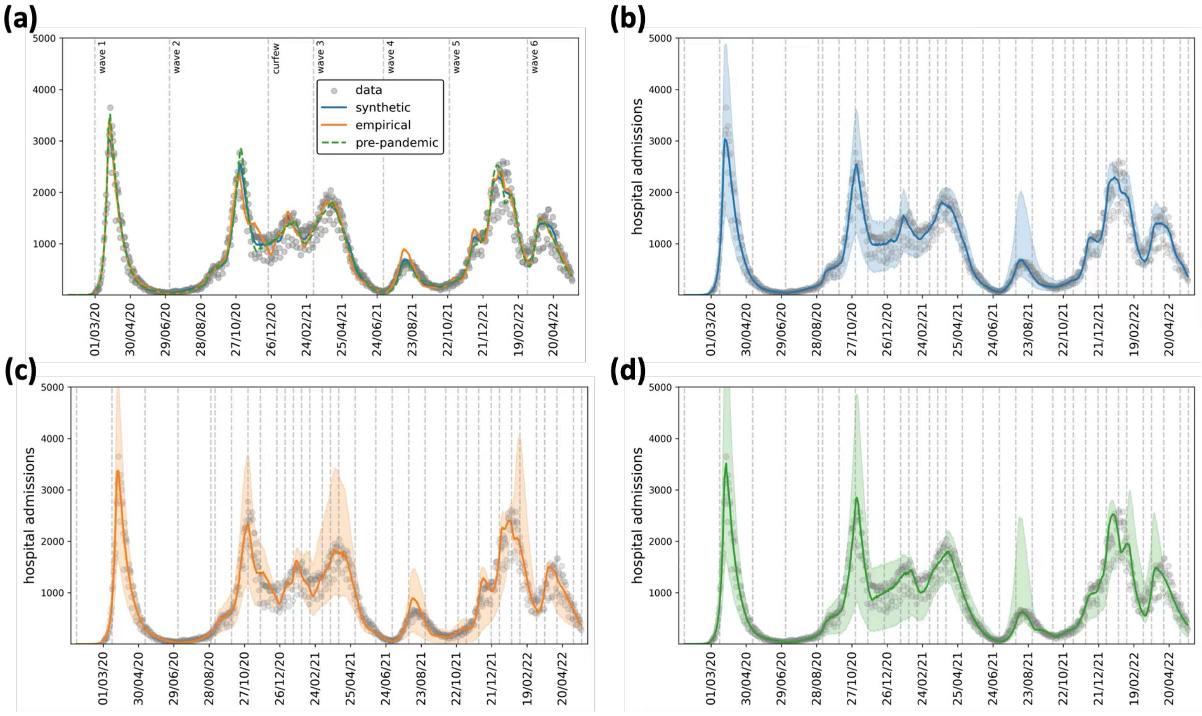

Figure S3. Model fit. (a) Results presented in **Fig. 5a** of the main text. Model median trajectory of daily hospital admissions (median values out of n=100 independent stochastic simulations), obtained by fitting the transmission model using weekly synthetic contact matrices (blue), extended empirical contact matrices (orange) and a constant pre-pandemic matrix (green). Data used for the fit are displayed with gray dots. Vertical dashed lines indicate different pandemic phases. **(b)** Daily number of hospital admissions predicted by the model using weekly synthetic contact matrices. Continuous line and shaded area indicate the median and 95% probability ranges respectively, computed over n=100 independent stochastic runs. The vertical dashed lines define the time-windows for the fit of the time-varying correcting factor (see **Table S8**). **(c-d)** As in panel (b), showing predictions of the model using the survey-based contact matrices in (c) and a constant pre-pandemic contact matrix in (d).

In **Table S9**, we report the values of goodness of fit in terms of Akaike Information Criterion (AIC). The AIC was computed based on the log-likelihood (see **Section 6**) of the model median trajectory with respect to the observed data. We found that, both overall and for each epidemic wave, the model informed with weekly synthetic contact matrices exhibits a higher goodness of fit (lower AIC) compared to the model using empirical contact matrices, suggesting that using the synthetic matrices allows to better capture disease-relevant changes of behavior over time and therefore better reproduce the epidemic trajectory.

Table S9. Goodness of fit. Values of AIC for the three models. The AIC was computed as $2k - 2\log(L)$ where L is the likelihood of the predicted median model trajectory compared to the data (overall or in a specific pandemic phase), and k is the number of parameters estimated, i.e. the number of correcting factors (overall or in the pandemic phase under consideration). The periods are defined as follows: overall (from 2020-03-01 to 2022-05-22), wave 1 (from 2020-03-01 to 2020-07-05), wave 2 (from 2020-07-06 to 2020-12-20), curfew (from 2020-12-21 to 2021-03-07), wave 3 (from 2021-03-08 to 2021-07-04), wave 4 (from 2021-07-05 to 2021-10-24), wave 5 (from 2021-10-25 to 2022-03-06), wave 6 (from 2022-03-07 to 2022-05-22).

	**AIC**							
**Model**	**Overall**	**Wave 1**	**Wave 2**	**Curfew**	**Wave 3 (Alpha)**	**Wave 4 (Delta)**	**Wave 5 (Delta and Omicron BA.1)**	**Wave 6 (Omicron BA.2)**
Synthetic	67040	7796	11473	7112	7289	5632	20787	6951
Empirical	95755	9435	19558	13013	10849	9053	26365	7452
Pre-pandemic	70647	7654	13091	7560	6575	8180	19024	8563

We note that, during periods marked by gradual changes in social behavior — such as school closures, curfews, and gradual lifting of restrictions — the model using synthetic contact matrices more accurately captured the trajectory of hospital admissions. For example, during the interval between the second wave and the onset of the third ("wave 2" to "curfew", **Fig. S4**), only the synthetic matrix model accurately reproduced the decline and subsequent resurgence in hospitalizations. In contrast, both the empirical and pre-pandemic matrix models produced discontinuities or "jumps" in the epidemic curve, reflecting unrealistic abrupt shifts in the effective reproduction number. This pattern suggests that these matrices failed to track gradual behavioral and policy-driven shifts in contact patterns, which are essential for realistic simulation of transmission dynamics.

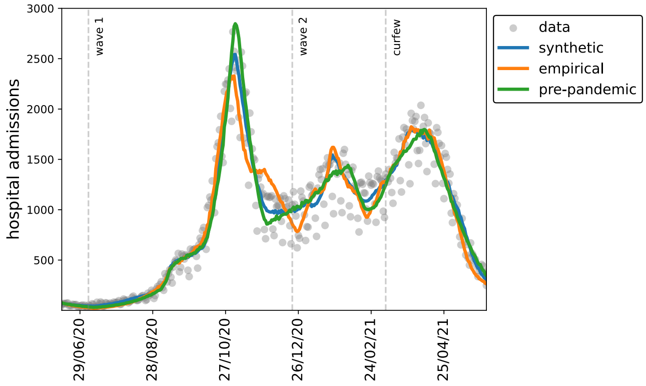

Figure S4. Model fit between the second and the third wave. Model median trajectory of daily hospital admissions (median values out of n=100 independent stochastic simulations), obtained by fitting the transmission model using weekly synthetic contact matrices (blue), extended empirical contact matrices (orange) and a constant pre-pandemic matrix (green). Data used for the fit are displayed with grey dots.

7.2 Correcting factor

In **Fig. 6a** in the main text, we showed the distribution of the correcting factor overall the study period (March 2020 – May 2022). Here we show the distribution broken down by pandemic phases or waves (**Fig. S5**). Pandemic phases are defined as follows: wave 1 (from 2020-03-01 to 2020-07-05), wave 2 (from 2020-07-06 to 2020-12-20), curfew (from 2020-12-21 to 2021-03-07), wave 3 (from 2021-03-08 to 2021-07-04), wave 4 (from 2021-07-05 to 2021-10-24), wave 5 (from 2021-10-25 to 2022-03-06), wave 6 (from 2022-03-07 to 2022-05-22). We further divide each wave into the rising phase and the declining phase.

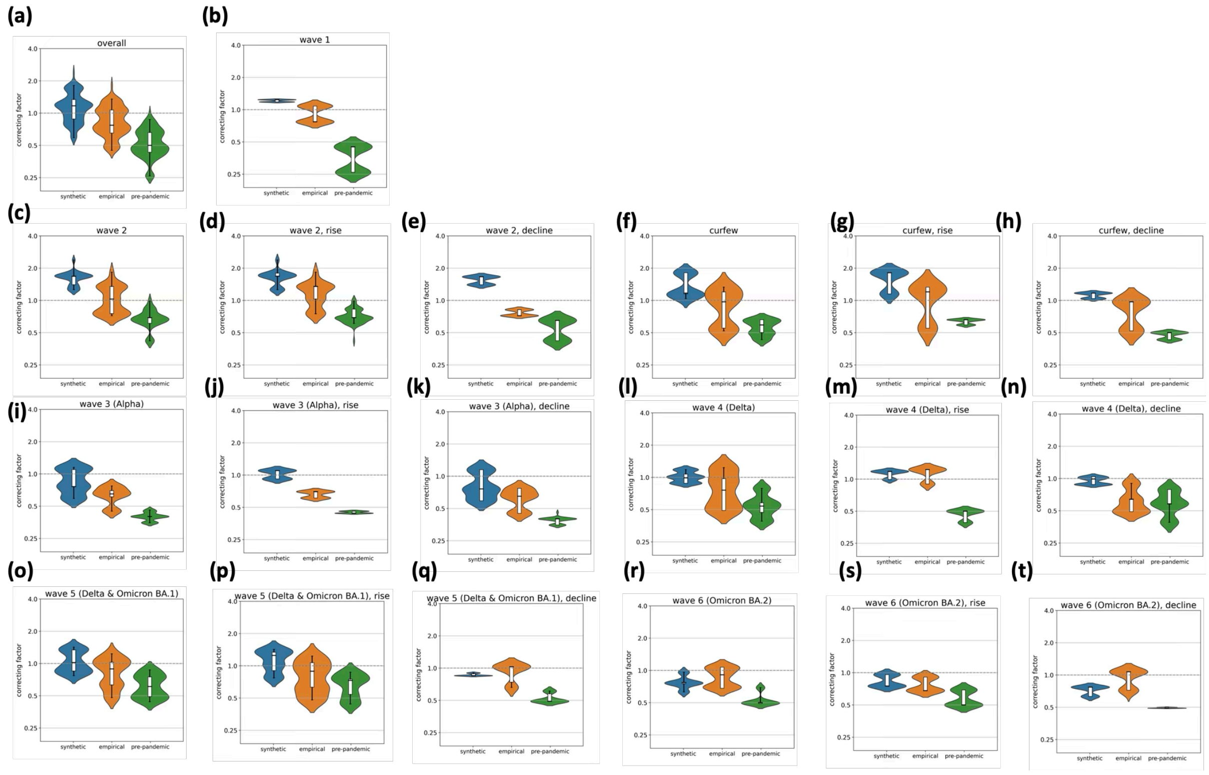

Figure S5. Distribution of the correcting factor by pandemic phase. Distribution of the correcting factor, fitted with the model using synthetic matrices (synthetic, blue), survey-based matrices (empirical, orange), and a constant pre-pandemic matrix (pre-pandemic, green). The box plot indicates median (line), interquartile range (box), and quantiles 2.5% and 97.5% (whiskers) of the values of the correcting factor from the start of the first lockdown to the end of the study period (March 2020 – May 2022, n=794 days). In all panels, the violin plots have been scaled in order to have the same maximum width, to enhance visual legibility; panel (a) is the same as **Fig. 6a** in the main text, except for the width adjustment. Panels a, b, c, f, i, l, o, r refer to the overall period and the 7 pandemic phases defined as in **Fig. 5a**. For each pandemic phase, we divided the distribution for the rising phase and the decline phase (panels d,e,g,h,j,k,m,n,p,q,s,t), except the first wave because in the rising phase of the first wave we do not fit a correcting factor. To distinguish between rising and declining phase, we used as threshold the date of the highest number of hospitalizations recorded in the period. The title of the panel indicates the pandemic phase under consideration.

We found some temporal correlation of the correcting factor across models (**Fig. S6**). The highest correlation was found for the models informed with the synthetic and empirical matrices (**Fig. S6a**). This suggests the presence of unmodeled factors — such as changes in testing, masking, or contact duration — that affect transmission potential independently of contact frequency.

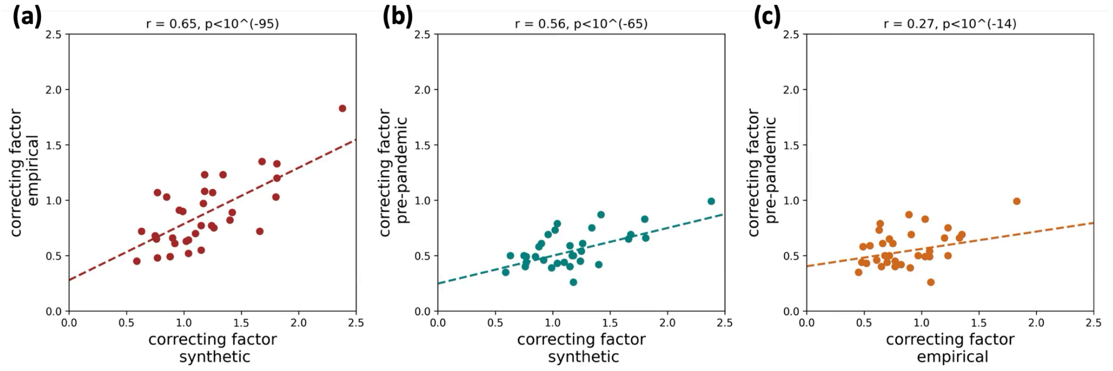

Figure S6. Correlation of correcting factor across models. (a) Scatter plot of the correcting factor for the model informed with synthetic (x axis) or empirical matrices (y axis). Values of r and p in the title of the panel represent the Pearson's correlation coefficient and p-value respectively. Dashed line is a linear fit, shown as guide for the eye. We use values of the correcting factor from the start of the first lockdown to the end of the study period (March 2020 – May 2022, n=794 days). The correcting factor follows a step-wise constant

function over n=32 fitting windows. **(b)** As in panel (a), for the model informed with synthetic matrices (x axis) or pre-pandemic matrix (y axis). **(c)** As in panel (a), for the model informed with empirical matrices (x axis) or pre-pandemic matrix (y axis).

7.3 HOSPITALIZATIONS BY AGE CLASS

In Fig. 5a and Fig. S3, we showed the model estimates for daily hospital admissions, aggregated by age group. Here, we show the model trajectories of hospital admissions by age group with the disaggregated hospital data.

For adults [19-64] and seniors 65+ y.o., we found that estimates of the model informed with synthetic contact matrices aligned with observations (relative variation -2.8% [-5.9, 3.2] in cumulative hospitalizations for adults; -0.7% [-1.5, 0.1] for seniors; **Fig. S8a**). Instead, the model informed with empirical contact matrices generated more hospitalizations in the adult age group and less in the senior age group, compared to the data (relative variation +5.8% [2.1, 7.7] for adults; -5.5% [-9.7, -2.3] for seniors; **Fig. S8a**), corresponding in absolute terms to approximately 13,000 additional hospitalizations in adults and 23,000 fewer hospitalizations in seniors (**Fig. S8b**). Both models (synthetic and empirical) produced an excess in hospitalizations compared to observations for the younger age groups (children [0-10] y.o. and adolescents [11-18] y.o.). The discrepancy was more evident in the model informed with empirical contact matrices (+147% [143, 151], +33% [30, 37] relative variation for children and adolescents, respectively) rather than the model with synthetic contact matrices (+106% [102, 114], +16% [11, 23]).

The model informed with the pre-pandemic matrix produced age-stratified hospitalizations that were closer to those obtained with the synthetic matrices than with the empirical ones. Still, the model informed with the pre-pandemic matrix produced on average lower hospitalizations in seniors and higher hospitalizations in adults, compared to the observed data and the synthetic model (**Fig. S8a,b**).

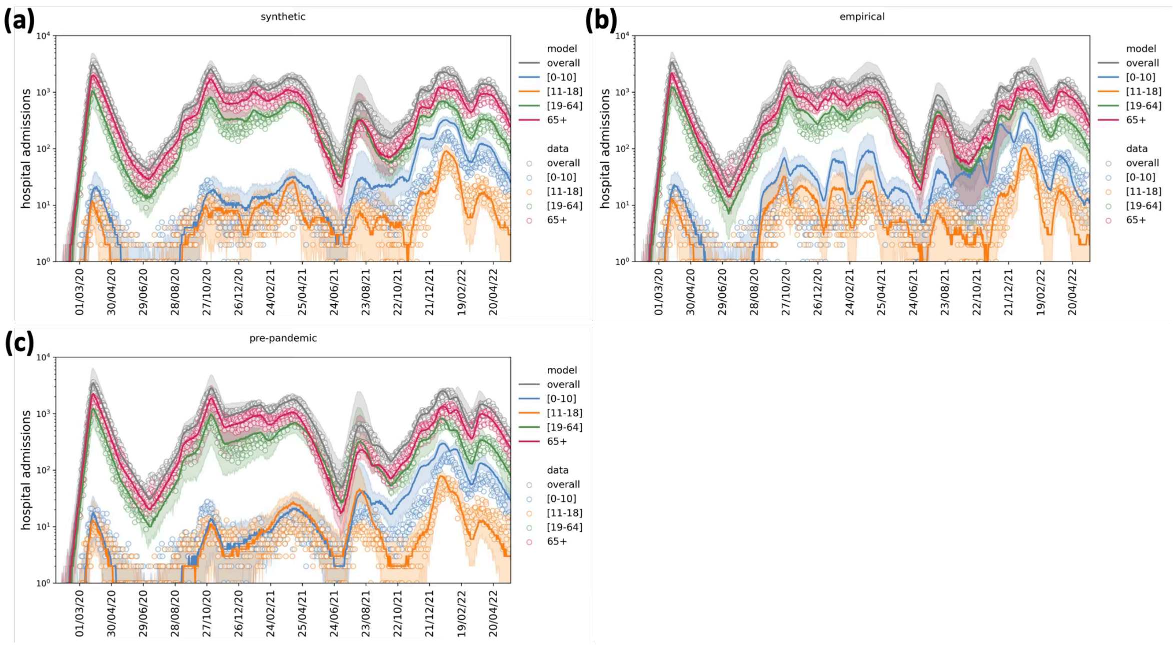

Figure S7. Trajectory of hospital admissions by age class. Top row: results of the model using weekly synthetic contact matrices. Bottom row: results of the model using survey-based contact matrices. **(a)** Model predictions obtained using weekly synthetic contact matrices. The model is fitted on aggregated numbers (gray line and dots). Trajectories by age class (colored lines) are shown in comparison with hospital data by age class (colored dots). Continuous line and shaded area indicate the median and 95% probability ranges respectively, computed over n=100 independent stochastic runs. **(b-c)** As in panels (a), showing results for the model using survey-based contact matrices (b) or a constant pre-pandemic contact matrix.

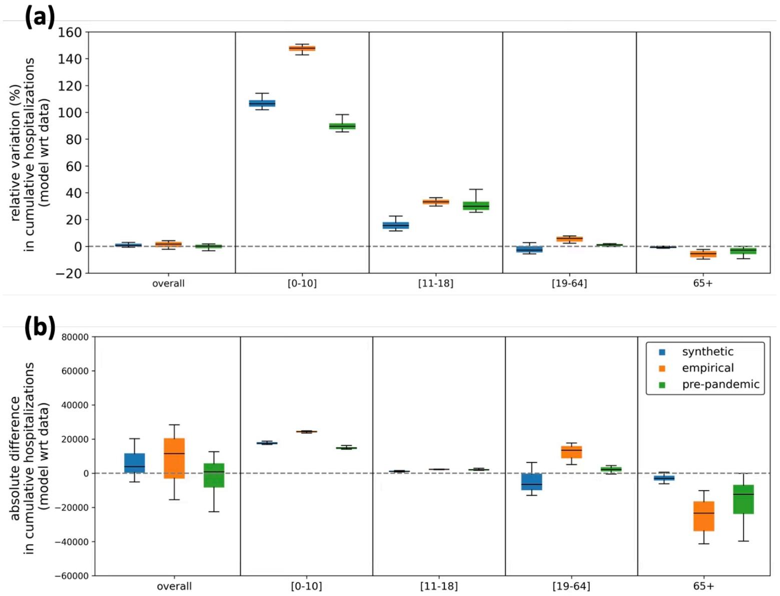

Figure S8. Comparison of model predictions with hospital admission data by age class. (a) Relative variation (overall and by age class) of the cumulative number of hospital admissions (from March 1, 2020 to May 22, 2022) predicted by the three models with respect to observations. **(b)** Absolute difference (overall and by age class) of the cumulative number of hospital admissions (from March 1, 2020 to May 22, 2022) predicted by the three models with respect to observations. This panel is also presented in the main text with a shorter span on the y-axis (**Fig. S5d**). In both panels, the box plot indicates median (line), interquartile range (box), and 2.5% and 97.5% quantiles (whiskers) out of n=100 independent stochastic runs.

7.4 ATTACK RATE

In **Fig. S9**, we show the cumulative number of new first infections (i.e. people entering the compartment E), by age group. From the trajectory of new infections, we extracted the trajectory of antibody-positive individuals by modeling seroconversion and seroreversion (see Methods and **Fig.6 c-f**).

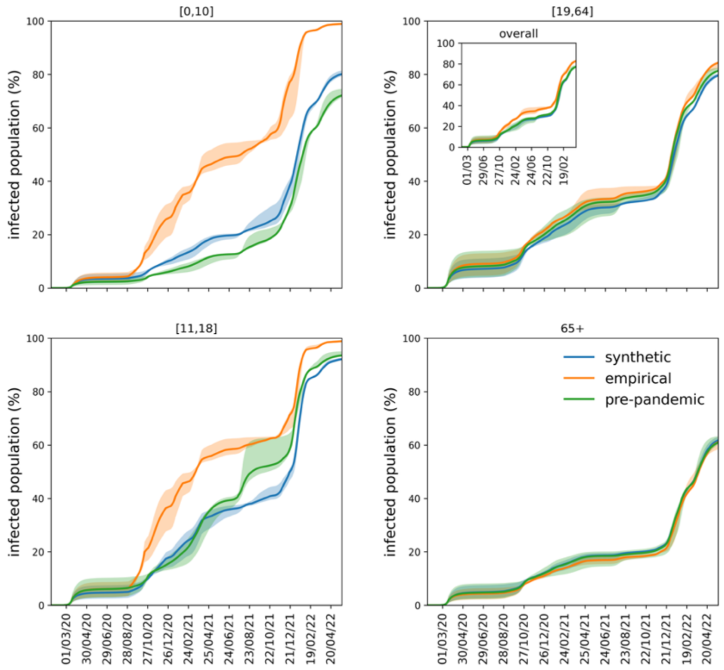

Figure S9. Cumulative number of (first) infections. Proportion of the cumulative number of first infections over time, estimated with the three models, by age class (from top-left, column-wise: children [0,10], adolescents [11-18], adults [19-64], seniors 65+), and overall (inset

in top-right panel). Lines and shaded areas indicate the median and 95% probability ranges, respectively, computed across n=100 stochastic simulations.

8. ADDITIONAL RESULTS ON COMPARISON OF CONTACT MATRICES

8.1 COMPARISON OF MIXING PATTERNS

In **Fig. S10**, we compare the matrices using different metrics, namely cosine similarity, overall assortativity and proportion of contacts generated by young individuals. Cosine similarity was higher during periods of school closure, indicating a larger agreement in age-specific mixing patterns between the two matrix sources (**Fig. 10a**). When schools were open, particularly in March 2021, similarity dropped, reflecting greater deviations in mixing among young age groups. Notably, cosine similarity between empirical and synthetic matrices was consistently higher than that between synthetic matrices and homogeneous mixing (i.e. a contact between any two individuals occur randomly with the same probability). Compared to the pre-pandemic contact matrix, synthetic matrices were slightly less assortative, while maintaining a similar proportion of contacts established by young individuals (around 25%, **Fig. S10b**). In contrast, empirical matrices showed larger differences with the pre-pandemic and synthetic contact matrices, with lower overall assortativity and a higher share of contacts generated by individuals under 19 years of age (about 50%, **Fig. S10b**).

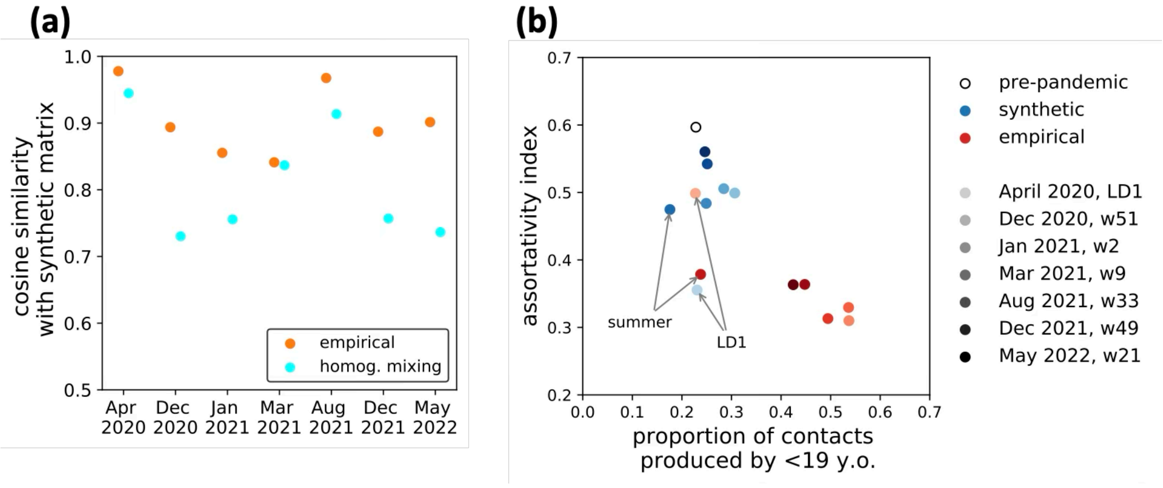

Figure S10. (a) Cosine similarity (invariant to global rescaling) between the synthetic and survey-based contact matrices, and between the synthetic matrices and matrices assuming homogeneous mixing. **(b)** Proportion of the overall connectivity produced by young individuals (<19 y.o.) vs age-assortativity index, in the empirical (reds) and synthetic matrices (blues), for the seven survey periods. The value for the pre-pandemic contact matrix (void black dot) is shown for reference.

8.2 CORRELATION WITH NORMALCY AND STRINGENCY INDEX

In **Fig. 3c** in the main text we showed the average number of contacts over time (as estimated in the synthetic matrices) together with the Normalcy Index. Here we show the Stringency Index (**Fig. S11a**). We computed the correlation between the average number of contacts (either in the synthetic or the SocialCov matrices) and the Stringency and Normalcy indexes (**Fig. S11b,c**). For both the synthetic and the empirical matrices, we found a high correlation with both the Stringency and the Normalcy index.

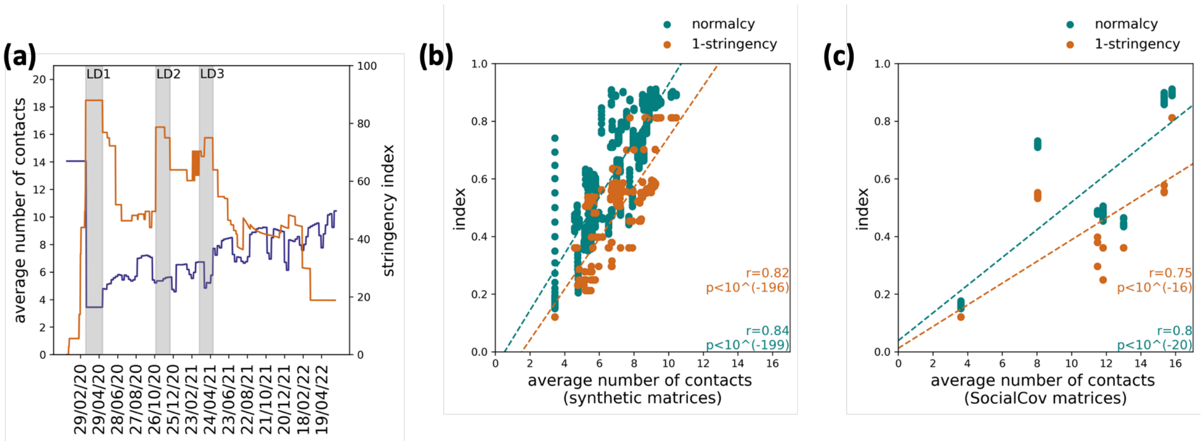

Figure S11. Correlation with Normalcy and Stringency Index. (a) Average number of contacts in the synthetic contact matrices (purple, left y-axis), compared with the stringency index over time (orange, right y-axis). Gray shaded areas represent the three lockdowns. **(b)** Scatter plot of the average number of contacts in the synthetic matrices and the corresponding stringency (orange) and normalcy (teal) index. The value of normalcy and stringency index are rescaled in between 0 and 1, and the plot shows the value of 1 – stringency, to align it with the interpretation of the normalcy index (the higher the index, the closer to pre-pandemic conditions). Values of r and p represent the Pearson's correlation coefficient and p-value respectively. Dashed line is a linear fit, shown as guide for the eye. We consider n=804 daily values from March 21st, 2020 to May 31st, 2022. **(c)** As in panel (b), considering the average number of contacts in the SocialCov matrices. The correlation is restricted to the seven survey periods.

8.3 Comparison with CoMix matrices

Besides the SocialCov survey, another study called CoMix collected social contact data during the COVID-19 pandemic, in multiple European countries. In France, the CoMix study carried out 7 survey waves for adults (from A1 to A7), and 2 survey waves for children, C1 and C2 (below 18 years of age). The calendar of the CoMix survey waves is detailed in **Table S10** and illustrated in **Fig. S12** in comparison with the SocialCov survey periods. We did not include this source of empirical matrices into our transmission model due to their limited temporal resolution (only spanning from late December 2020 to April 2021). However, in this section we report the analysis of contact patterns in CoMix compared to synthetic and SocialCov matrices. CoMix data for France were available on Zenodo[61].

Table S10. Periods of data collection and survey participants in the CoMix study in France.

Survey wave	Survey participants	Survey period
A1	Adults	December 21-29, 2020
A2	Adults	January 7-12, 2021
A3	Adults	January 20-22, 2021
C1	Children (< 18 y.o.)	February 3-9, 2021
A4	Adults	February 17-20, 2021
A5	Adults	March 3-8, 2021
A6	Adults	March 17-20, 2021
C2	Children (< 18 y.o.)	April 2-8, 2021
A7	Adults	April 14-15, 2021

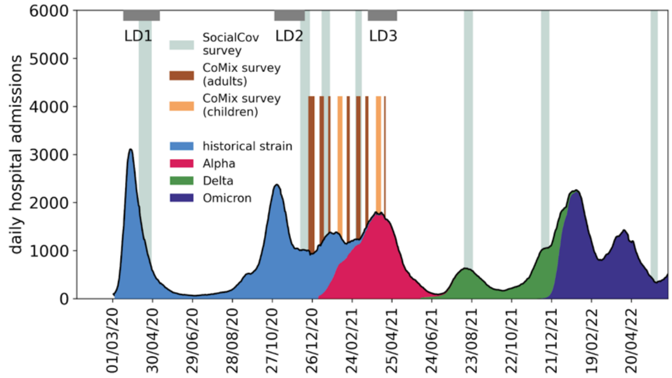

Figure S12. Periods of data collection for SocialCov and CoMix surveys.

We estimated 2 CoMix contact matrices, by pulling together the results of the closest adult-child survey waves (therefore, we merged A4-C1 and A7-C2 to estimate a full contact matrix). We considered only contacts on regular weekdays (Monday-Friday excluding holidays), to ensure comparability with our main analysis. We compared the resulting CoMix matrices with the weekly synthetic matrices for the week corresponding to the CoMix children wave (i.e., February 2021 (w5) and April 2021 (w13)) and with the closest SocialCov matrices (i.e., January 2021 and March 2021). The matrices are illustrated in **Fig. S13**.

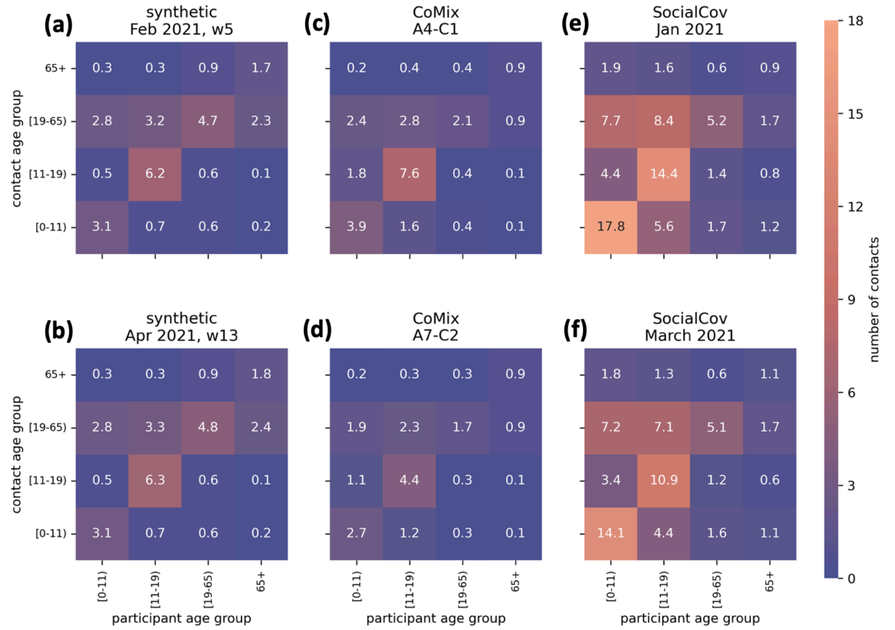

Figure S13. Illustrations of contact matrices. In panels a,b, two synthetic contact matrices corresponding to week 5 and week 13 in 2021. In panels c,d, CoMix contact matrices estimated by merging the survey waves A4-C1 and A7-C2. In panels e,f, SocialCov matrices for January and March 2021, the closest to the CoMix survey waves for comparison.

We computed the average number of contacts, overall and by age group. We found that participants in the CoMix survey reported substantially fewer contacts compared to those in the SocialCov survey (**Fig. S14a**). The average number of contacts was around ⅓ in the CoMix matrices compared to SocialCov matrices (**Fig. S14c**). Fewer contacts were reported in all age groups (**Fig. S14d-g**), and the strongest difference was measured in children (around ¼, **Fig. S14c**). Compared to synthetic matrices, the number of contacts in CoMix was lower in adults and seniors, and comparable in children and adolescents (**Fig. S14b**).

We also computed the four metrics of comparison (cosine similarity, assortativity, young connectivity and age-specific fraction of within-group contacts). The cosine similarity between the CoMix matrices and the two other sources (either synthetic or SocialCov matrices) was comparable (**Fig. S14l**). Contacts in the CoMix matrices were substantially more assortative than SocialCov matrices, and they were more similar to synthetic matrices in terms of overall assortativity and age-specific proportion of within-group contacts (**Fig. S14h-k**, **Fig. S14m**). However, CoMix and SocialCov matrices were similar in terms of young connectivity (around 50%) in contrast with synthetic contact matrices (around 25%, **Fig. S14m**).

In conclusion, our analysis showed that CoMix matrices reported substantially fewer contacts than SocialCov, particularly in children, while their structure was more similar to synthetic matrices in terms of assortativity and age-specific within-group mixing. Still, young individuals contributed to overall connectivity in CoMix matrices at levels comparable to SocialCov and higher than in synthetic matrices.

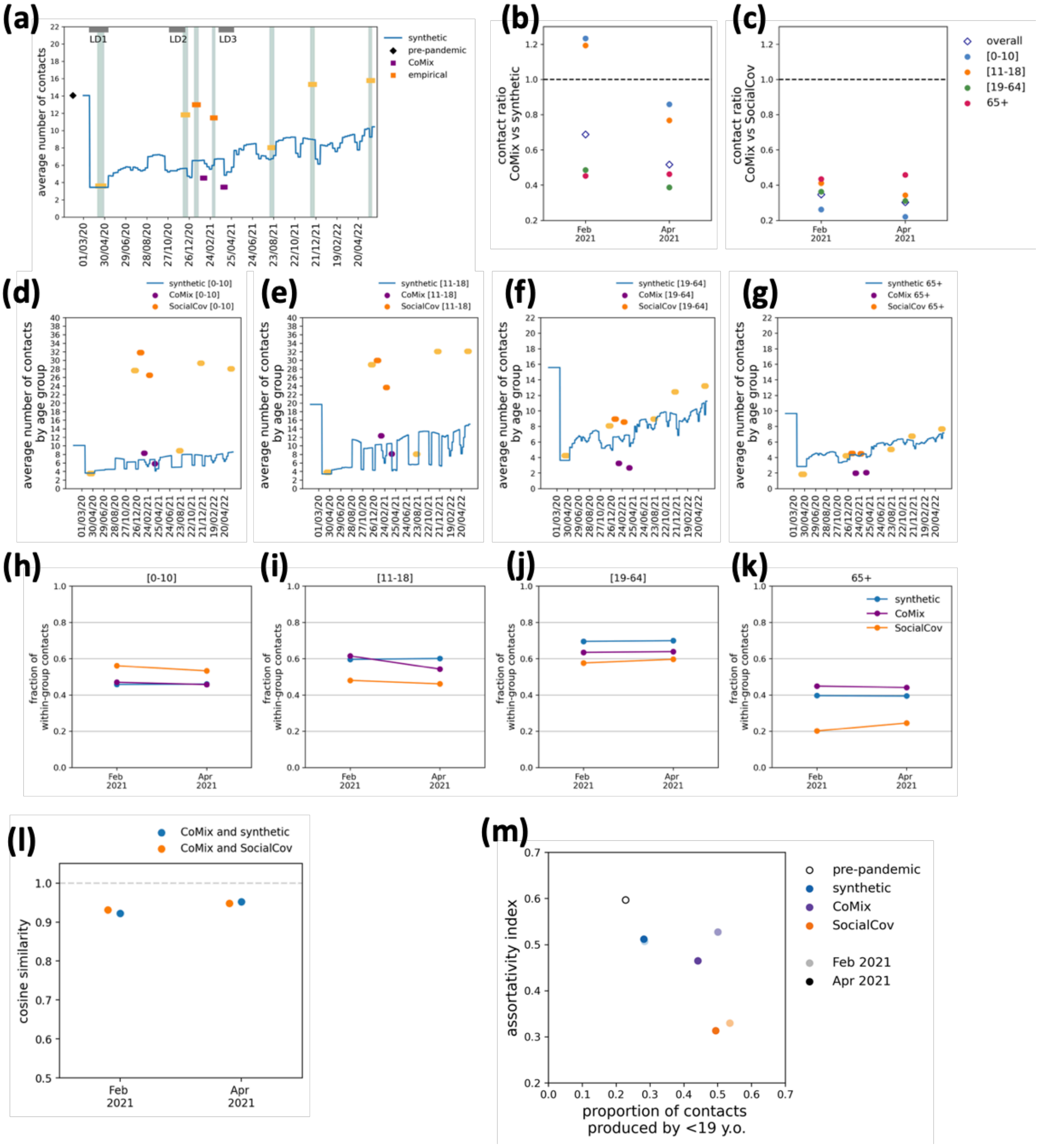

Figure S14. Comparison of contact volumes and matrix structure in the CoMix, synthetic and SocialCov matrices. (a) Average number of contacts over time, in the synthetic matrices (blue line), in the two SocialCov contact matrices under consideration (orange; the other SocialCov matrices are shown as comparison in yellow), and in the two estimated coMix matrices (purple). The value of the pre-pandemic empirical contact matrix used for baseline is shown in black. **(b)** Ratio of the number of contacts estimated in the CoMix matrices with

respect to the synthetic matrices, broken down by survey wave (x-axis) and by age group (filled dots) or overall (void diamonds). **(c)** Ratio of the number of contacts estimated in the CoMix matrices with respect to the empirical SocialCov matrices, broken down by survey wave (x-axis) and by age group (filled dots) or overall (void diamonds). **(d-g)** Average number of contacts over time by age groups (from left to right, children, adolescents, adults and seniors), in the synthetic matrices (blue line), in the two SocialCov contact matrices under consideration (orange; the other SocialCov matrices are shown as comparison in yellow), and in the two estimated coMix matrices (purple). **(h-k)** Fraction of within-group contacts for each age group (from left to right, children, adolescents, adults and seniors), defined as $M_{ii}/\sum_j M_{ij}$, in the synthetic matrices (blue), SocialCov matrix (orange) and CoMix matrices (purple). **(l)** Cosine similarity (invariant to global rescaling) between the CoMix and synthetic contact matrices (blue), and between the CoMix and SocialCov matrices (orange). **(m)** Proportion of the overall connectivity produced by young individuals (<19 y.o.) vs age-assortativity index, in the two CoMix matrices (purple) and in the two synthetic (blue) and SocialCov matrices (orange) chosen for comparison. The value for the pre-pandemic contact matrix (void black dot) is shown for reference.

9. SENSITIVITY ANALYSES

9.1 Specification of empirical contact matrices

In this section, we present the results of two sensitivity analyses regarding the definition of the survey-based contact matrices and their integration in the transmission model. Results of the sensitivity analyses are displayed in **Fig. S15**.

The proportion of infections and therefore antibody-positive individuals in the young age groups ([0-10] and [11-18]) predicted by the model using the survey-based contact matrices (reported in the main analysis in **Fig. 6**) could be exaggerated due to the fact that we did not account for the weekend effect (and therefore school closure) in the contact matrices. For this reason, we carried out a sensitivity analysis where we informed the model using weighted empirical matrices, where participant weights were assigned based on the day of the survey, either a weekday or a weekend. As described in Ref.[62], to align the survey data to this distribution, one can obtain participant weights in the form of $w = (5/7)/(N_{weekday}/N)$ OR $(2/7)/(N_{weekend}/N)$, where N is the sample size, and $N_{weekday}$ and $N_{weekend}$ are the number of participants that were surveyed during weekdays and weekend days, respectively. After re-fitting the model using the weighted empirical contact matrices, we found that the estimated proportion of infections and antibody-positive population in the young age groups is lower compared to the main analysis (**Fig. S15c,d**). The predictions are in line with the serological estimate for June 2021, but still not compatible with the serological estimate for February 2021 (**Fig. S15c,d**). The change in the definition of the SocialCov matrices leads to a slight improvement of the AIC and mean absolute error compared to the empirical model of the main analysis. However, the best model fit, both in terms of likelihood and mean absolute error, is still the model using the synthetic contact matrices (**Table S11**, **Fig. S15g**).

We also tested a model where we used the survey-based matrix estimated for May 2022 as a pre-pandemic contact matrix. This was done in order to use a pre-pandemic matrix that had a similar structure and distribution of contacts, rather than the original pre-pandemic matrix which is more similar to the synthetic matrices (by construction). This matrix specification led to an increase in seroprevalence in young age groups during the first wave and summer 2020 (**Fig. S15c,d**). We also note that, with this matrix specification, the AIC improves slightly with respect to the survey-based model presented in the main analysis (**Table S11**), however the mean absolute error is worse. This discrepancy may be due to the fact that AIC and MAE quantify different aspects of model accuracy. AIC is computed from the likelihood, which reflects how well the model explains the overall data distribution. A model with the better AIC might be giving a better fit to the data's distribution even if the absolute deviations are higher, while a model with lower MAE might be doing well in minimizing large deviations, but it may not reflect the distribution of the data as accurately. In any case, both the MAE and the AIC lean towards the model informed with synthetic matrices.

Table S11. Goodness of fit. Values of AIC for the synthetic and empirical models of the main analysis, and the for the two models with alternative specifications of the survey-based matrices, considered for sensitivity analysis.

Model	AIC
Synthetic	67040
Empirical	95755
Sensitivity weekend	91393
Sensitivity pre-pdm matrix	93396

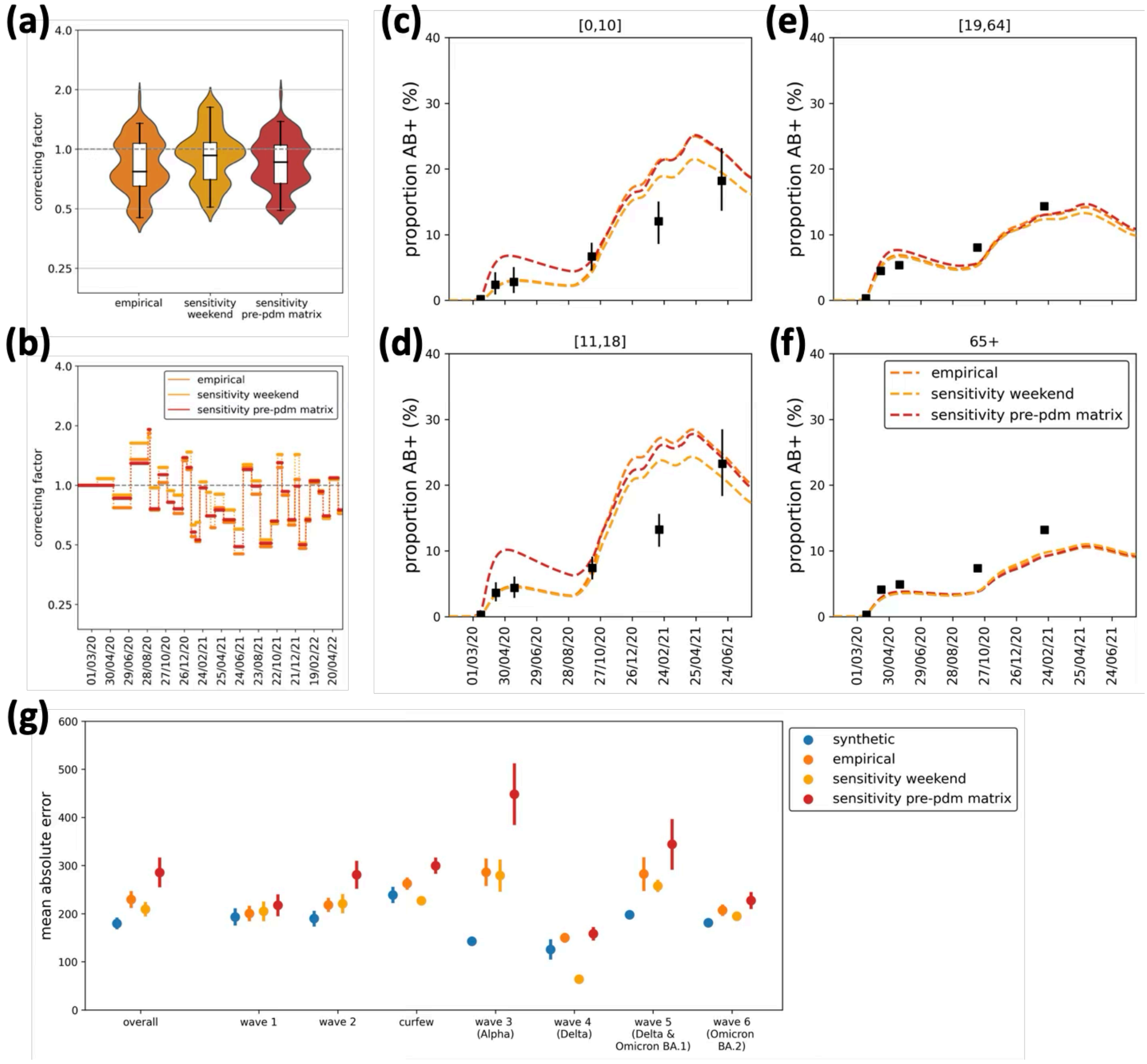

Figure S15. Sensitivity on the model using empirical contact matrices. In all panels, 'synthetic main' and 'empirical main' indicates the results of the main text presented in **Fig. 5,6**; 'sensitivity weekend' indicates the results obtained informing the model with empirical contact matrices adjusted for weekday/weekend; 'sensitivity pre-pdm matrix' indicates the results obtained using the empirical matrix of May 2022 as pre-pandemic matrix. **(a)** Distribution of the correcting factor in the three models. The box plot indicates median (line), interquartile range (box), and quantiles 2.5% and 97.5% (whiskers) of the values of the correcting factor from the start of the first lockdown to the end of the study period (March 2020 – May 2022, n=794 days). **(b)** Correcting factor over time. **(c-f)** Proportion of antibody-positive population over time, estimated with the three models, by age class (from top left, column-wise: [0,10], [11-18], [19-64], 65+). Black symbols indicate estimates from serological data[63]. Dashed lines indicate the median computed across n=100 independent stochastic simulations. **(g)** Mean absolute error (MAE) of daily model predictions with respect to the daily observed data, on the overall

period (March 2020 – May 2022) and broken down by epidemic phase (epidemic waves and in-between periods). Dots and lines represent the average MAE and 95% confidence interval computed across n=100 stochastic runs.

9.2 Relative susceptibility of young individuals

In the main analysis, the relative susceptibility of children and adolescents with respect to adults was set at 70% for the Wuhan strain, and to 100% for the variants (Alpha, Delta, Omicron). As a sensitivity analysis, we tested a relative susceptibility of 70% for all the variants (i.e. the same as for the Wuhan strain).

Even in this new parameterization, the model informed with mobility-based synthetic matrices has a better AIC than the empirical and pre-pandemic model (**Table S12**), in line with the conclusion of main analysis. In terms of seroprevalence, using a lower susceptibility leads to a decrease in the predicted antibody-positive population in young individuals, in all models; the discrepancy in the trajectories becomes visible from the end of February onwards, i.e. after the take-over of the Alpha variant.

Table S12. Values of AIC for the three models, assuming 100% relative susceptibility (reference) or 70% relative susceptibility (sensitivity).

	**AIC**	
**Model**	**Reference Susceptibility**	**Susceptibility tested for sensitivity**
Synthetic	67040	61258
Empirical	95755	88542
Pre-pandemic	70647	63259

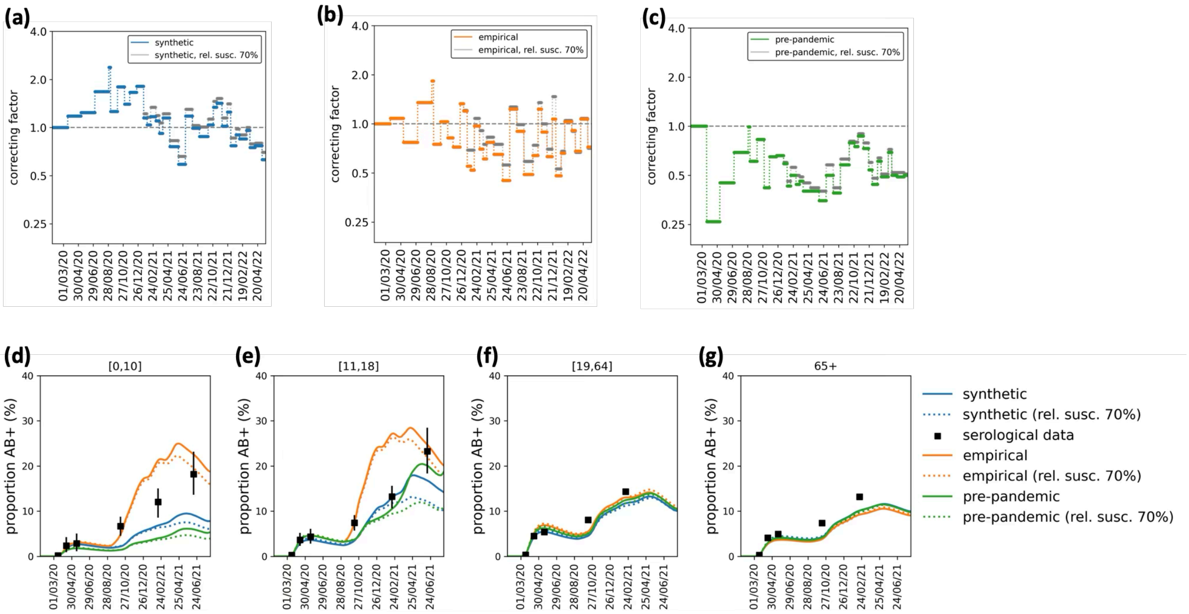

Figure S16. Impact of assuming a lower susceptibility for young individuals relative to adults with the variants. (a) Correcting factor over time, fitted with the model informed with synthetic matrices, using the parameterization in the main analysis (colored line) or in the sensitivity analysis (grey line). **(b)** As in panel (a), showing results of the sensitivity analysis for the model informed with empirical matrices. **(c)** As in panel (a), showing results of the sensitivity analysis for the model informed with a constant pre-pandemic matrix. **(d-g)** Proportion of antibody-positive population over time, estimated with the three models, by age class (from left to right: children [0,10], adolescents [11-18], adults [19-64], seniors 65+). Black symbols indicate estimates from serological data[63]. Lines indicate the median computed across n=100 independent stochastic simulations. Continuous lines indicate the results of the main analysis (**Fig. 6c-f**) and dashed lines indicate the results of the sensitivity analysis.

9.3 CALIBRATION TARGETS

In the main analysis, we fitted the transmission model to the age-stratified number of hospital admissions per day, and also age-stratified serological data (**Fig. 5a**). We performed a sensitivity analysis using only hospitalization data for the fit (either age-stratified, or total counts only), to assess potential changes in the estimated correcting factor and relative performance of the models.

Results are shown in **Fig. S17-S21**. The results are robust to these alternative specifications of the likelihood. No significant variation is observed in the resulting correcting factors or in the mean absolute errors. The model built on synthetic matrices continues to be preferred model in terms of AIC (**Table S13**), in line with the conclusions of the main analysis.

The results of this sensitivity analysis indicate that our conclusions do not depend on the specific choice of calibration inputs. Notably, incorporating serological data did not materially alter the performance of the synthetic matrices, suggesting that these matrices remain informative for real-time use even in settings where serological surveillance is absent.

Table S13. Goodness of fit. Values of AIC for the three models (synthetic, empirical and pre-pandemic), using different calibration targets. For a given calibration target, a model with a lower AIC indicates a higher goodness of fit.

	**AIC**		
**Model**	**Fit on age-stratified hospital data and serological data**	**Fit on age-stratified hospital data**	**Fit on overall hospital data**
Synthetic	67040	67420	27624
Empirical	95755	94992	34356
Pre-pandemic	70647	69353	34690

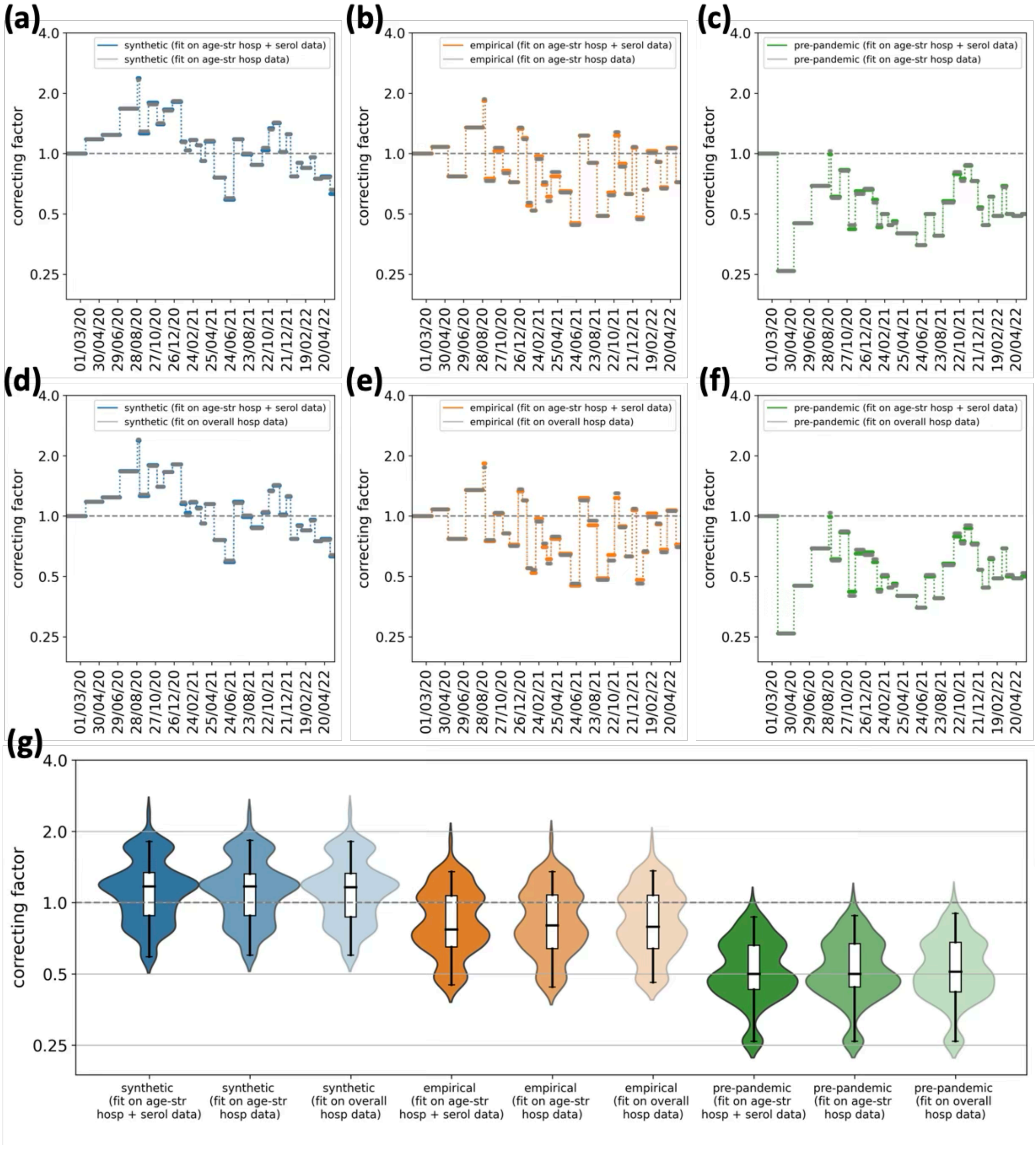

Figure S17. Sensitivity of correcting factor using other calibration targets. (a-c) Comparison of correcting factor over time, fitted on age-stratified hospital and serological data (colored line) or fitted on age-stratified hospital data only (grey line). Results obtained using the model informed with synthetic matrices (panel a), empirical matrices (panel b), and a static pre-pandemic matrix (panel c). **(e-f)** Comparison of correcting factor over time, fitted on age-stratified hospital and serological data (colored line) or fitted on overall hospital data only (grey line). Results obtained using the model informed with synthetic matrices (panel d), empirical matrices (panel e), and a static pre-pandemic matrix (panel f). **(g)** Distribution of the correcting factor in the three models, for each calibration target (fit on age-stratified hospital data and serological data; fit on age-stratified hospital data only; fit on overall hospital data). The box plot indicates median (line), interquartile range (box), and quantiles 2.5% and 97.5% (whiskers) of the values of the correcting factor from the start of the first lockdown to the end of the study period (March 2020 – May 2022, n=794 days).

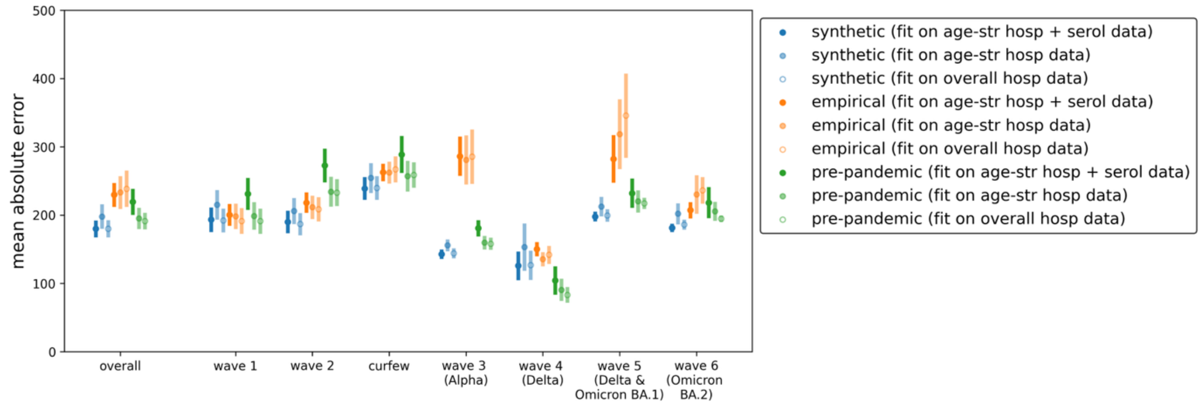

Figure S18. Sensitivity of mean absolute error using other calibration targets. Mean absolute error (MAE) of daily model predictions with respect to the daily observed data, on the overall period (March 2020 – May 2022) and broken down by epidemic phase (epidemic waves and in-between periods). Dots and lines represent the average MAE and 95% confidence interval computed across n=100 stochastic runs. Shades of color indicate the results using the three calibration targets (fit on age-stratified hospital data and serological data; fit on age-stratified hospital data only; fit on overall hospital data).

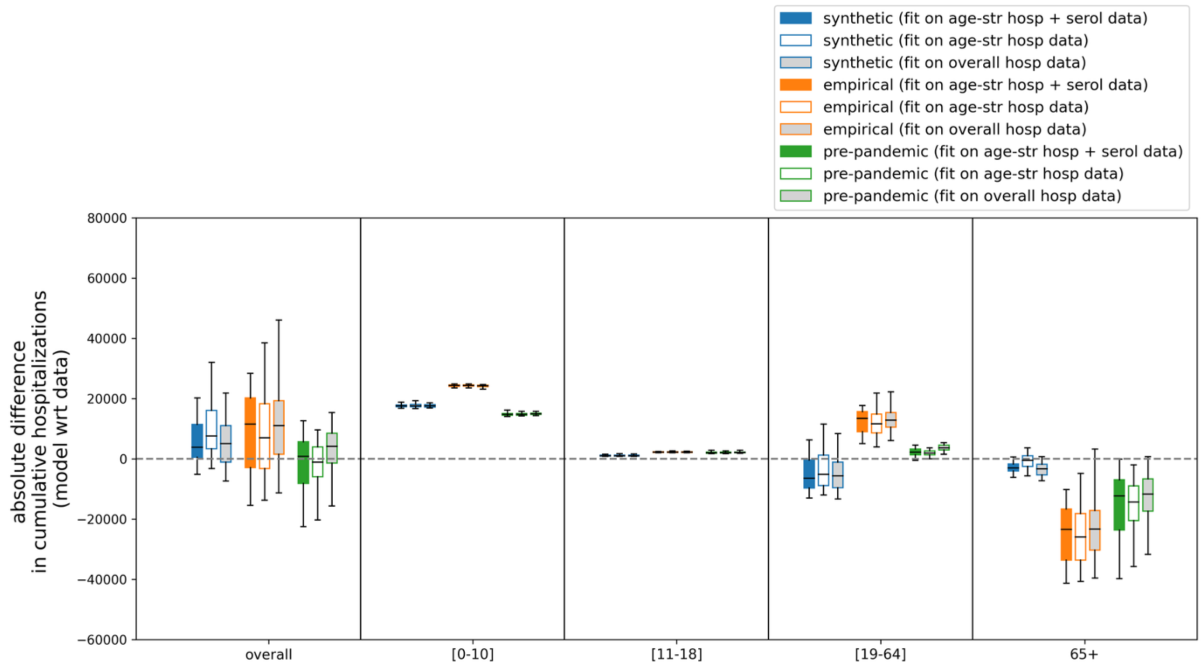

Figure S19. Sensitivity in the cumulative number of predicted hospitalizations, using other calibration targets. The plot shows the absolute difference (overall and by age class) of the cumulative number of hospital admissions (from March 1, 2020 to May 22, 2022) predicted by the three models, compared to observations. Results are shown for the three calibration targets (fit on age-stratified hospital data and serological data; fit on age-stratified hospital data only; fit on overall hospital data). The box plot indicates median (line), interquartile range (box), and 2.5% and 97.5% quantiles (whiskers) out of n=100 independent stochastic runs. Box plots filled in color indicate the results of the main analysis, while boxplots filled in white or grey refer to the sensitivity analyses.

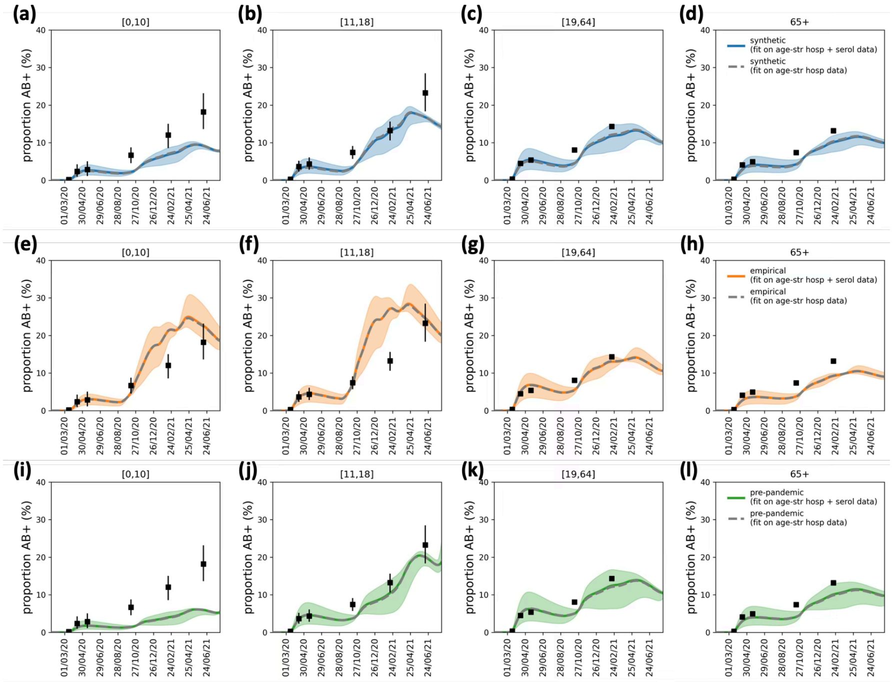

Figure S20. Sensitivity in the proportion of antibody-positive population, fitting on age-stratified hospitalizations. (a-d) Proportion of antibody-positive population over time, estimated with the model using the fit of the main analysis (colored line, fit on age-stratified hospital and serological data) or using a different calibration target (grey line, fit on age-stratified hospital admission only). Each panel shows the results for one age group (from left to right, [0,10], [11-18], [19-64], 65+). Black symbols indicate estimates from serological data. Results refer to the transmission model informed with synthetic matrices. **(e-h)** As in panels a-d, showing results of the transmission model informed with empirical matrices. **(i-l)** As in panels a-d, showing results of the transmission model informed with a static pre-pandemic matrix. In all panels, lines and shaded areas indicate respectively the median and 95% probability ranges (2.5% and 97.5% quantiles) computed across n=100 independent stochastic simulations.

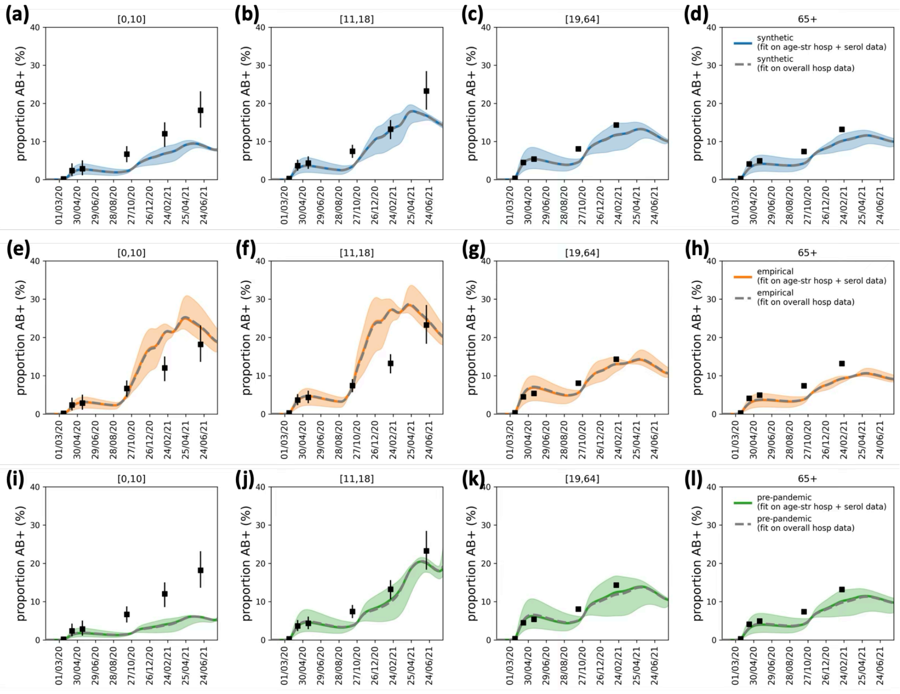

Figure S21. Sensitivity in the proportion of antibody-positive population, fitting on age-stratified hospitalizations. (a-d) Proportion of antibody-positive population over time, estimated with the model using the fit of the main analysis (colored line, fit on age-stratified hospital and serological data) or using a different calibration target (grey line, fit on age-stratified hospital admission only). Each panel shows the results for one age group (from left to right, [0,10], [11-18], [19-64], 65+). Black symbols indicate estimates from serological data. Results refer to the transmission model informed with synthetic matrices. **(e-h)** As in panels a-d, showing results of the transmission model informed with empirical matrices. **(i-l)** As in panels a-d, showing results of the transmission model informed with a static pre-pandemic matrix. In all panels, lines and shaded areas indicate respectively the median and 95% probability ranges (2.5% and 97.5% quantiles) computed across n=100 independent stochastic simulations.

9.4 VARIATIONS IN THE TOTAL NUMBER OF CONTACTS PER AGE GROUP

We tested the role of the synthetic contact matrix in informing who is most at-risk, via the total number of contacts per age group. To this purpose, we generated a modified time-varying contact matrix, derived from the original synthetic contact matrix by altering the average number of contacts per age group, while preserving the total number of contacts in the population at each time step. Specifically, we applied variations in the range observed in empirical data: at each time step, we reduced the average number of contacts engaged by adults by 67% and increased those in the elderly proportionally to ensure that the total number of contacts in the population matched that of the original synthetic matrix (see **Fig. S22c,d**). We then corrected the matrices for reciprocity, resulting in slight variations in the number of contacts for children and adolescents (around 10%, **Fig. S22a,b**). We refer to this set of matrices as TEST1 (adults+, seniors-). The properties (fraction of within group contacts, similarity, assortativity and young connectivity) of this set of contact matrices are shown in **Fig. S22e-j** in comparison with the synthetic contact matrices.

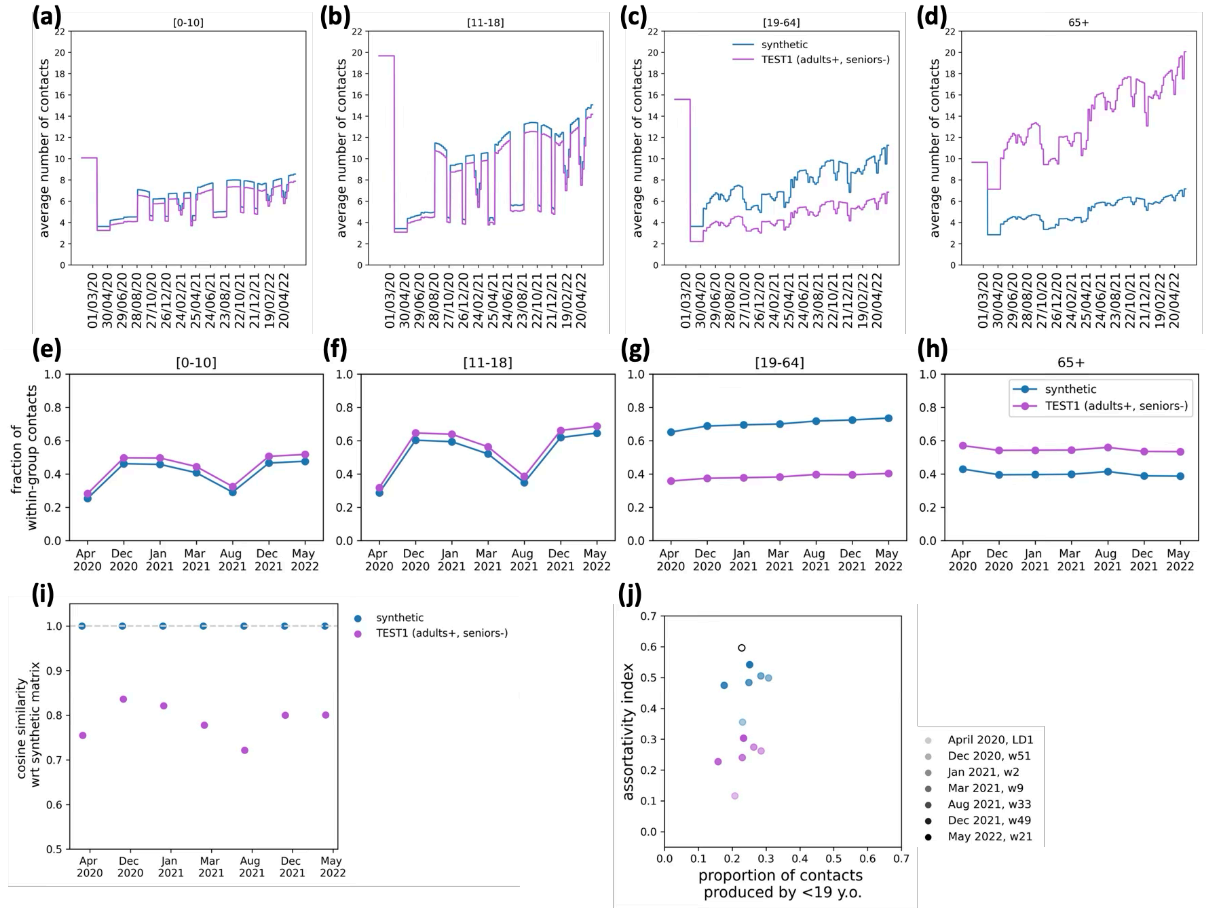

Figure S22. Comparison of matrices in TEST1 (adults+, seniors-) and the original synthetic matrices. (a-d) Total number of contacts over time, by age group (children in (a), adolescents in (b), adults in (c), seniors in (d)), in the synthetic matrices (blue) and in the matrices of TEST1 (purple). **(e-h)** Fraction of within-group contacts, for children (e), adolescents (f), adults (g) and seniors (h). **(i)** Cosine similarity between the matrices of TEST1 and the synthetic contact matrices. **(j)** Proportion of the overall connectivity produced by young individuals (<19 y.o.) vs age-assortativity index, in the synthetic matrices (blue), and in the matrices of TEST1 (purple), for the seven survey periods. The value for the pre-pandemic contact matrix (void black dot) is shown for reference.

We then fitted the transmission model using this new sequence of matrices. The results are illustrated in **Table S14** and in **Fig. S23-S24**. The correcting factor was lower than with the original synthetic matrices, but still close to 1 (median and IQR 0.87 [0.65 - 1.0] for TEST 1 and 1.17 [0.88 - 1.34] for the original model, **Fig. S23a,b**). The mean absolute error on the full trajectory of total hospitalizations was higher than the original model, with discrepancies visible especially in the Omicron phase (**Fig. S23c**). Substantial deviations emerged

in age-specific outcomes: hospitalizations among adults and seniors deviated significantly from observed data under TEST1 (**Fig. S24a**). TEST1 also led to lower predicted seroprevalence in adolescents and adults relative to serological data (**Fig. S24c,d**). In terms of model fit, the test model performed worse than the original synthetic model according to the Akaike Information Criterion (**Table S14**).

The deviations of age-stratified model outcomes from observations underscore that the role of the contact matrix in identifying who is most at risk through group-specific contact rates has epidemic consequences that cannot be offset by adjusting a global correcting factor. This demonstrates that (1) the correcting factor is not a reliable indicator of contact matrix performance and (2) it cannot absorb structural distortions in age-specific contact volumes. The time series of contacts per age group in the synthetic matrices is therefore essential.

Table S14. Goodness of fit. Values of AIC for the model using synthetic matrices or matrices of TEST1.

**Model**	**AIC**
Synthetic	27624
TEST1 (adults+, seniors-)	273237

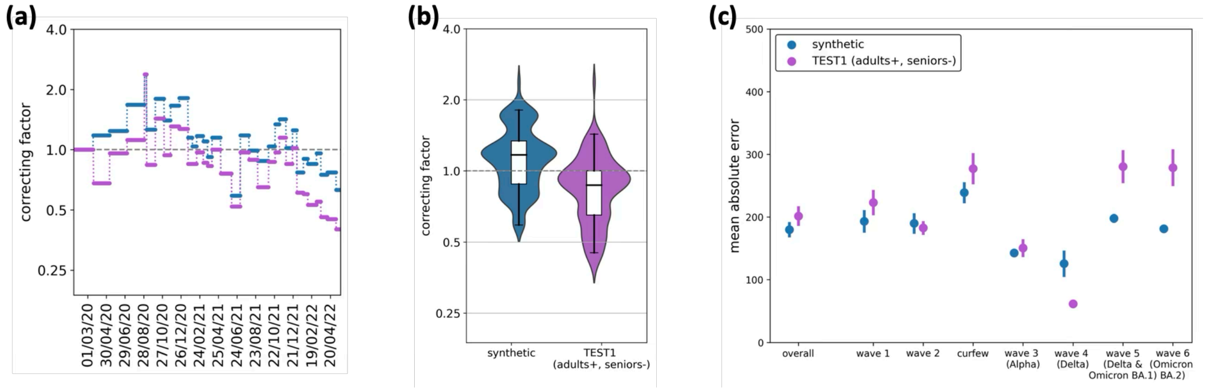

Figure S23. Sensitivity of correcting factor and model error using synthetic matrices or TEST1 matrices. (a) Correcting factor over time, estimated for the transmission model using synthetic matrices (in blue) or the modified contact matrices (TEST1, in purple). **(b)** Distribution of the correcting factor in the two models. The box plot indicates median (line), interquartile range (box), and quantiles 2.5% and 97.5% (whiskers) of the values of the correcting factor from the start of the first lockdown to the end of the study period (March 2020 – May 2022, n=794 days). **(c)** Mean absolute error (MAE) of daily model predictions with respect to the daily observed data, on the overall period (March 2020 – May 2022) and broken down by epidemic phase (epidemic waves and in-between periods). Dots and lines represent the average MAE and 95% confidence interval computed across n=100 stochastic runs.

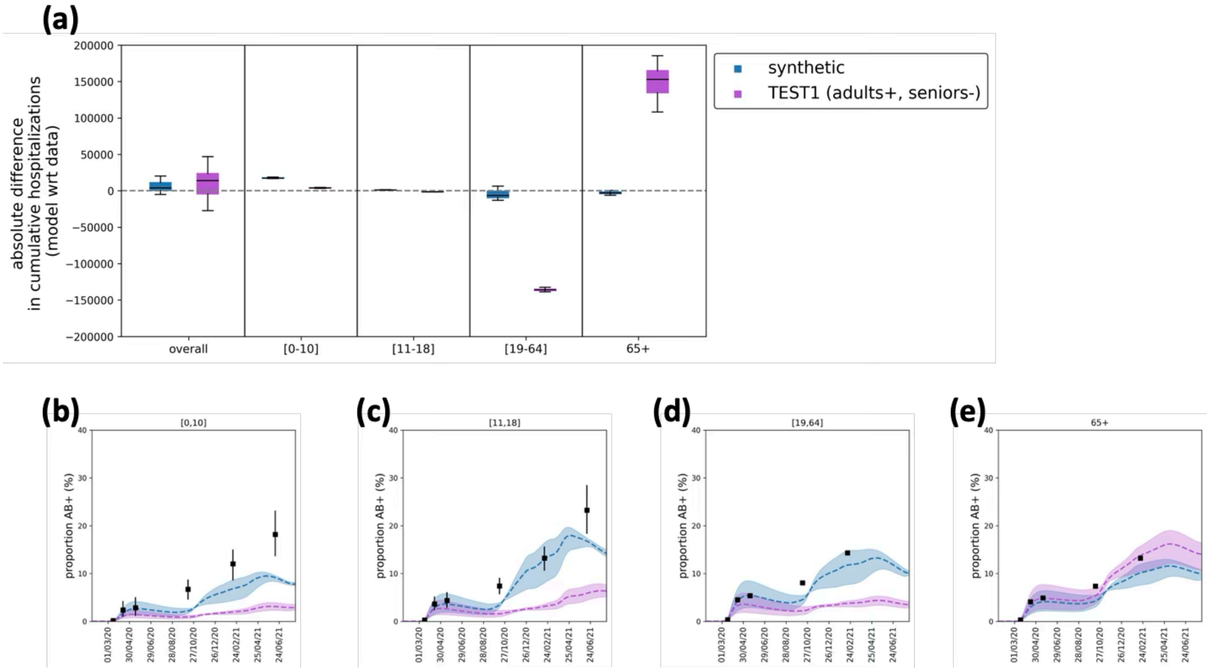

Figure S24. Sensitivity of age-stratified model outcomes using synthetic matrices or TEST1 matrices. (a) Absolute difference (overall and by age class) of the cumulative number of hospital admissions (from March 1, 2020 to May 22, 2022) predicted in the two models (synthetic in blue, and TEST1 in purple), compared to observations. Notice that, compared to analogous panels in other figures, here the y-axis range is extended by one order of magnitude. The box plot indicates median (line), interquartile range (box), and 2.5% and 97.5% quantiles (whiskers) out of n=100 independent stochastic runs. **(b-e)** Proportion of antibody-positive population over time, by age class (from left to right, [0,10], [11-18], [19-64], 65+). Black symbols indicate estimates from serological data. Lines and shaded areas indicate respectively the median and 95% probability ranges (2.5% and 97.5% quantiles) computed across n=100 independent stochastic simulations.

9.5 Variations in mixing patterns

To assess the impact of non-random mixing across age groups, we carried out two additional tests where we did not alter the total number of contacts per age group (this factor was previous investigated in Section 9.4), but we altered the distribution of the contacts across the alter age group. In TEST2 (random mixing), we generated a sequence of time-varying contact matrices defined where the total number of contacts of a given age group are preserved, but redistributed proportionally to the contacts offered by each other group. In practice, let $X_i = N_i \sum_j M_{ij}$ being the total number of contacts offered by group i, with M_{ij} being the original synthetic contact matrix. We computed $X_{ij} = (Xi \otimes Xi) / \sum_i Xi$, where $\otimes$ indicates the outer product of the two vectors, and defined a random mixing matrix as $\widehat{M}_{ij} = X_{ij}/N_i$. We introduced also TEST3 (less assortative), where we altered the mixing structure by reducing within-group contacts by 25% — consistent with empirically observed variations — and reallocating the resulting surplus to between-group contacts, proportionally to contact rates. This approach allowed us to reduce assortativity while preserving the total number of contacts per age group (up to maximum 15% of variation after adjustments for reciprocity). The comparison of the contact matrices of TEST2 (random mixing) and TEST3 (less assortative) with the original synthetic matrices is illustrated in **Fig. S25**.

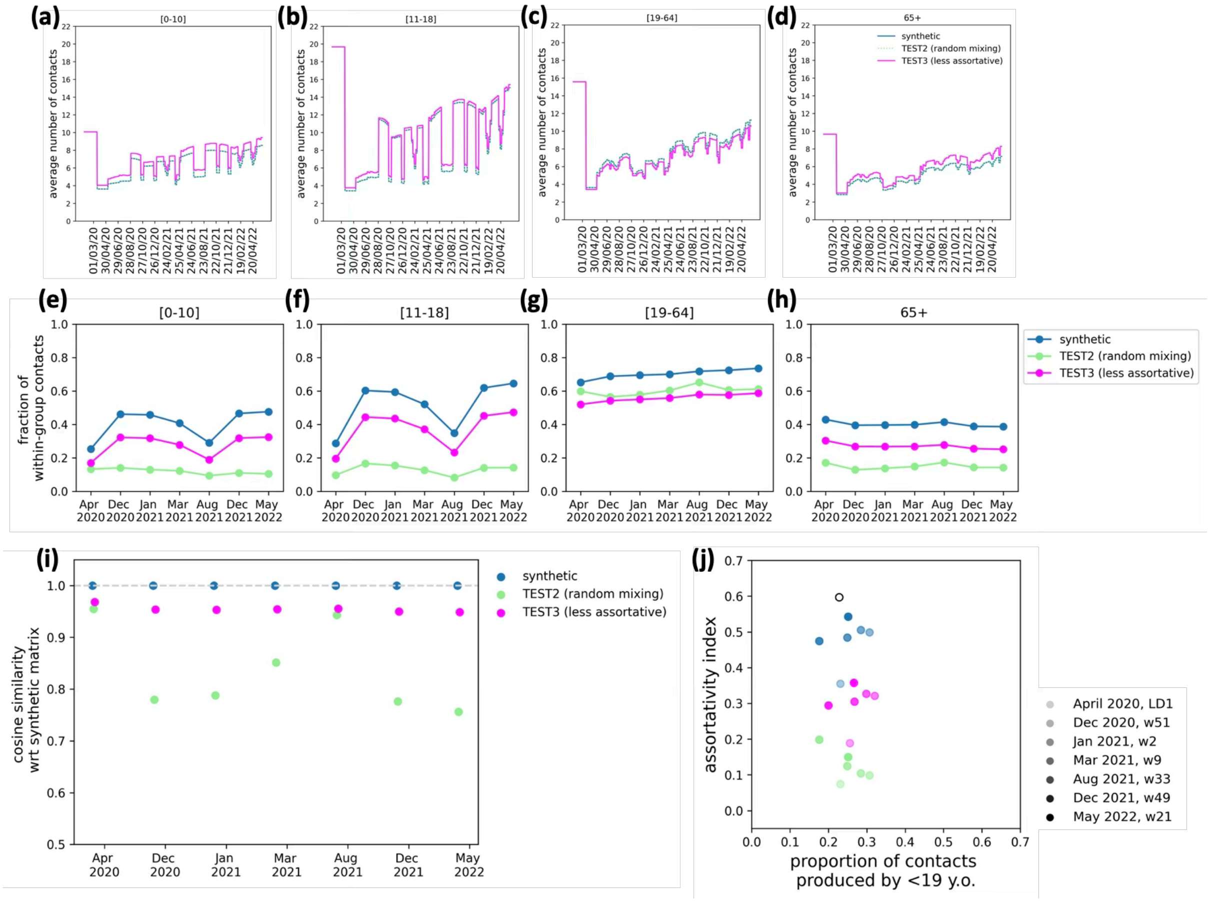

Figure S25. Comparison of matrices in TEST2 and TEST3 with the original synthetic matrices. (a-d) Total number of contacts over time, by age group (children in (a), adolescents in (b), adults in (c), seniors in (d)), in the synthetic matrices (blue) and in the matrices of TEST2 (light green, equivalent to the synthetic matrices) and TEST3 (magenta, where slight variations are due to the reciprocity adjustments). **(e-h)** Age-specific proportion of within-group contacts (from left to right, [0,10], [11-18], [19-64], 65+). **(i)** Cosine similarity between the matrices of TEST2 or TEST3, and the synthetic contact matrices. **(j)** Proportion of the overall connectivity produced by young individuals (<19 y.o.) vs age-assortativity index, in the synthetic matrices (blue), and in the matrices of TEST2 (light green) and TEST3 (magenta). The value for the pre-pandemic contact matrix (void black dot) is shown for reference.

We then fitted the transmission model using these new sequences of matrices, TEST2 (random mixing) and TEST3 (less assortative). The results are illustrated in **Table S15**, **Fig. S26-S27**. The correcting factor remained robust against the changes in the matrices introduced in TEST2 and TEST3 (**Fig. S26a-c**). The mean absolute error on total hospitalizations was comparable to that of the original model for TEST3 in all pandemic phases, while TEST2 produced markedly higher errors during the Omicron wave (**Fig. S26d**). However, substantial deviations emerged in age-specific outcomes. Both TEST2 and TEST3 produced higher hospitalizations in seniors and lower hospitalizations in adults (**Fig. S27a**). Both TEST2 and TEST3 led to lower predicted seroprevalence in adults and in adolescents relative to serological data (**Fig. S27c,d**). In terms of model fit, both test models performed worse than the original synthetic model according to the Akaike Information Criterion (AIC) (**Table S15**).

These results underscore the importance of the structure of age-specific mixing in shaping epidemic outcomes. Both tests altered the distribution of contacts across alter age groups, while preserving the total number of contacts per age group. Despite this constraint, the models still produced notable deviations from observed hospitalizations and serological data. Together, these findings demonstrate that it is not only the number of contacts per group that matters (as investigated in TEST1), but also from whom those contacts arise. The internal structure of the contact matrix — even when overall contact volumes are preserved — has substantial influence on both the magnitude and distribution of epidemic indicators. This reinforces the role of synthetic

contact matrices in capturing time-varying non-random mixing patterns as a fundamental component of realistic transmission modeling, beyond what a global scaling factor can adjust for.

Table S15. Goodness of fit of the model using synthetic matrices, matrices of TEST2 or matrices of TEST3.

Model	AIC
Synthetic	67040
TEST2	87442
TEST3	72359

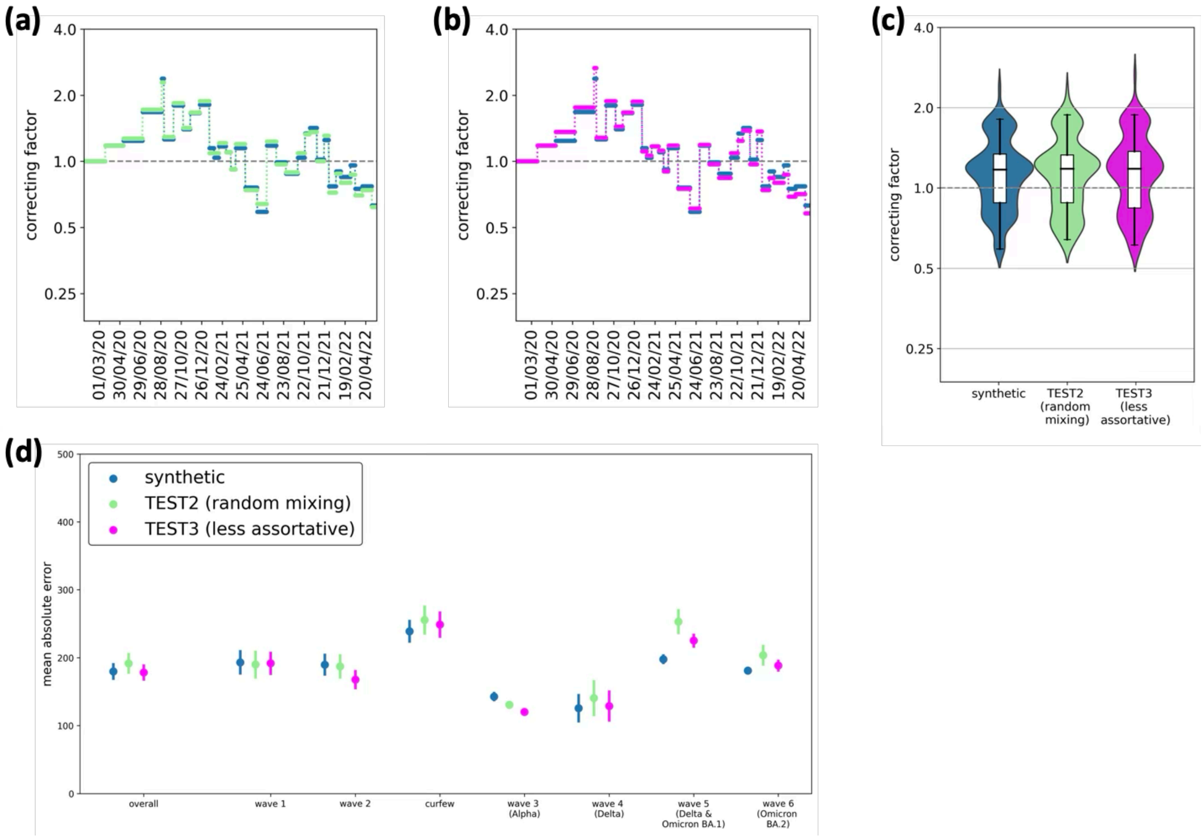

Figure S26. Sensitivity of correcting factor and model error using synthetic matrices, TEST2 matrices or TEST3 matrices. (a) Correcting factor over time, estimated for the transmission model using synthetic matrices (in blue) or the modified contact matrices (TEST2, in light green). **(b)** Correcting factor over time, estimated for the transmission model using synthetic matrices (in blue) or the modified contact matrices (TEST3, in magenta). **(c)** Distribution of the correcting factor in the three models. The box plot indicates median (line), interquartile range (box), and quantiles 2.5% and 97.5% (whiskers). **(d)** Mean absolute error (MAE) of daily model predictions with respect to the daily observed data, on the overall period (March 2020 – May 2022) and broken down by epidemic phase (epidemic waves and in-between periods). Dots and lines represent the average MAE and 95% confidence interval computed across n=100 stochastic runs.

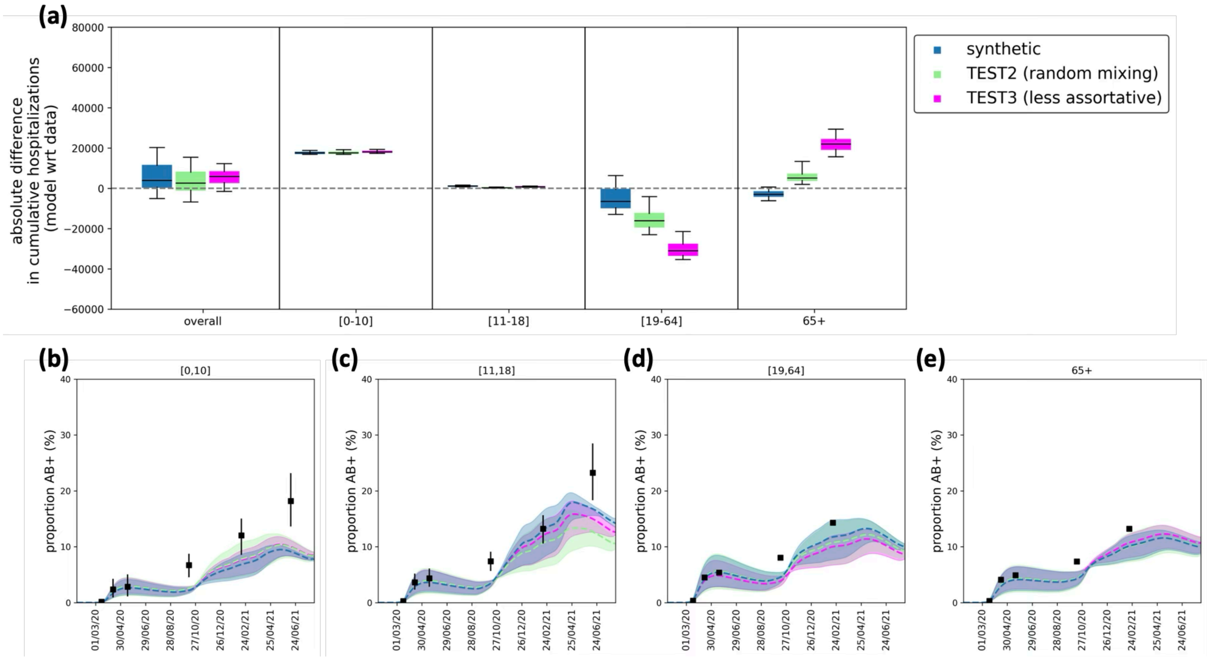

Figure S27. Sensitivity of age-stratified model outcomes using synthetic matrices, TEST2 matrices or TEST3 matrices. (a) Absolute difference (overall and by age class) of the cumulative number of hospital admissions (from March 1, 2020 to May 22, 2022) predicted in the three models (synthetic in blue, TEST2 in light green, TEST3 in magenta), compared to observations. The box plot indicates median (line), interquartile range (box), and 2.5% and 97.5% quantiles (whiskers) out of n=100 independent stochastic runs. **(b-e)** Proportion of antibody-positive population over time, by age class (from left to right, [0,10], [11-18], [19-64], 65+). Black symbols indicate estimates from serological data. Lines and shaded areas indicate respectively the median and 95% probability ranges (2.5% and 97.5% quantiles) computed across n=100 independent stochastic simulations.

9.6 IGNORING AVOIDANCE OF PHYSICAL CONTACTS

Since the survey data on physical contact avoidance[51] did not specify whether avoided physical contacts were replaced by non-physical ones, the model required a simplifying assumption. We chose to remove the avoided physical contacts, acknowledging this as a limiting — but clearly defined — lower-bound scenario. We did not assume replacement with non-physical contacts because we lack data to support such a transition, and because there is no evidence to parameterize different transmission rates for physical vs non-physical contacts. For this reason, we treated all contacts as equally transmissible.

We conducted a sensitivity analysis in which we ignored avoidance and retained all physical contacts (i.e., assuming either no avoidance or full replacement with equally transmissible non-physical contacts). We refer to this as TEST4 (keep phys contacts). As expected, this increased the average number of contacts across age groups (**Fig. S28a-e**). We then fitted the transmission model using the sequence of matrices from TEST4. Results from TEST4 were then compared with those from our main analysis (**Table S16**, **Fig. S29-S30**). We found that the model parameterized with the TEST4 matrices, while improving the fit of seroprevalence in adolescents relative to serological data in June 2021 (**Fig. S30c**), still worsened the fit for hospitalizations in seniors (**Fig. S30a**) and seroprevalence in children (**Fig. S30b**). Both the MAE and AIC indicated that TEST4 model performed better than the empirical (SocialCov) model, but still worse than the original synthetic model using contact avoidance (**Fig. S29c**, **Table S16**).

Overall, this analysis suggests that our original assumption provides a conservative yet effective approach, and confirms the robustness of our conclusions to different treatments of contact avoidance.

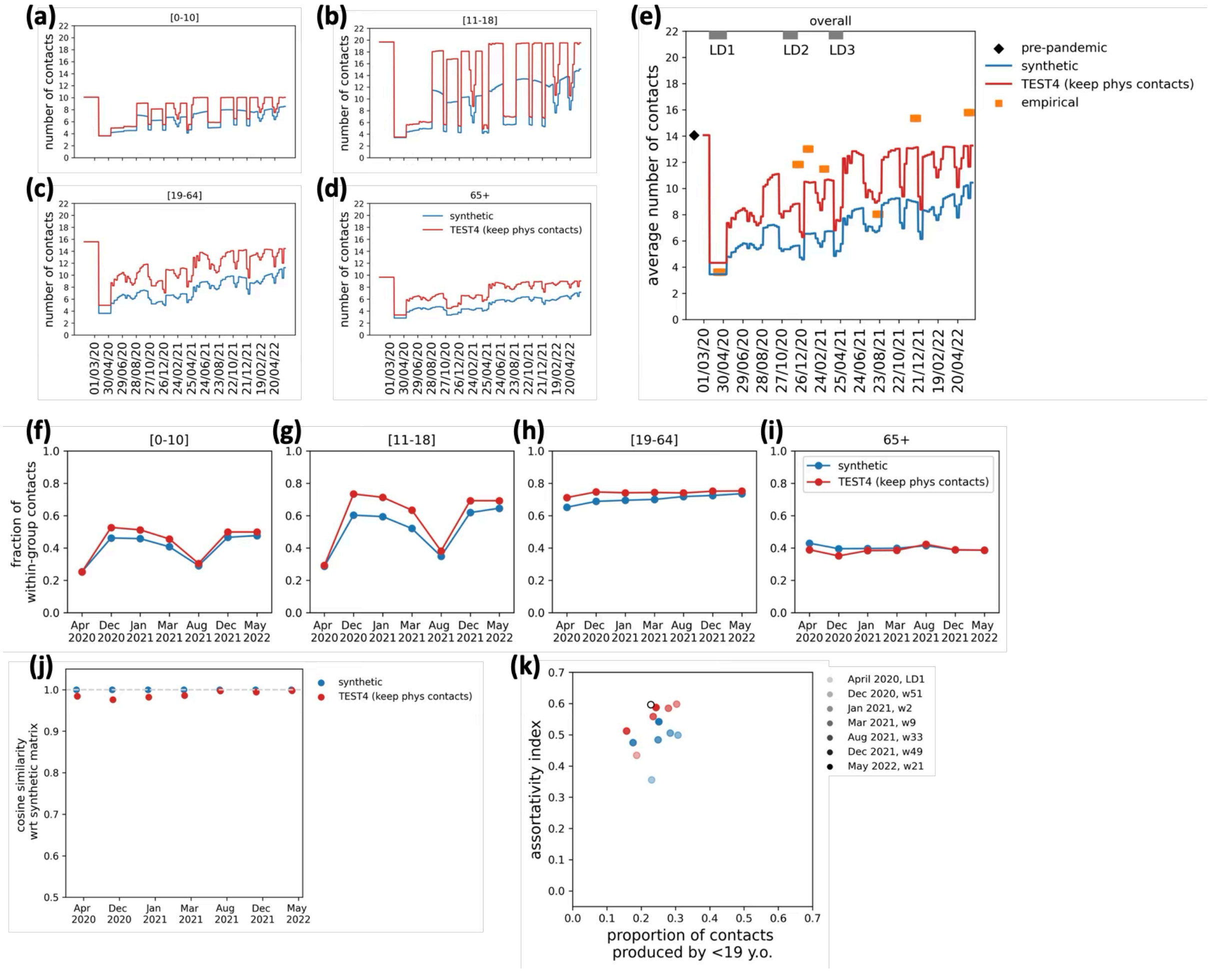

Figure S28. Comparison of matrices in TEST4 and the original synthetic matrices. (a) Total number of contacts in children over time, in the synthetic matrices (blue) and in the matrices of TEST4 (red). **(b)** As in panel a, for adolescents. **(c)** As in panel a, for adults. **(d)** As in panel a, for seniors. **(e)** Average number of contacts over time, in the synthetic (blue), TEST4 (red) and empirical contact matrices (orange). The value of the pre-pandemic empirical contact matrix used for baseline is shown in black. **(f-i)** Age-specific proportion of within-group contacts (from left to right, [0,10], [11-18], [19-64], 65+). **(j)** Cosine similarity between the matrices of TEST4 and the synthetic contact matrices. **(k)** Proportion of the overall connectivity produced by young individuals (<19 y.o.) vs age-assortativity index, in the synthetic matrices (blue), and in the matrices of TEST4 (red). The value for the pre-pandemic contact matrix (void black dot) is shown for reference.

Table S16. Goodness of fit of the model, using the synthetic, empirical, pre-pandemic matrices, or matrices in TEST4.

Model	AIC
Synthetic	67040
TEST4	69214
Empirical	95755
Pre-pandemic	70647

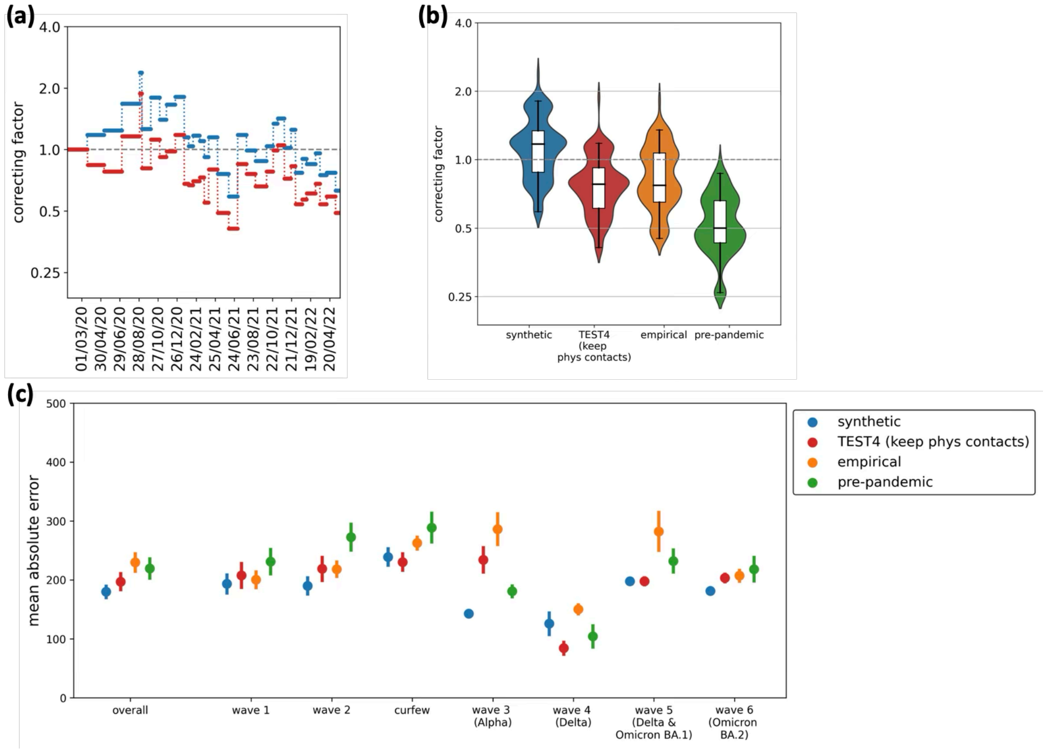

Figure S29. Sensitivity of correcting factor and model error using synthetic, empirical, pre-pandemic matrices, or matrices in TEST4. (a) Correcting factor over time, estimated for the transmission model using synthetic matrices (in blue) or TEST4 matrices (red). **(b)** Distribution of the correcting factor, estimated for the transmission model using synthetic matrices (in blue), TEST4 matrices (in red), empirical matrices (orange) and pre-pandemic matrix (green). The box plot indicates median (line), interquartile range (box), and quantiles 2.5% and 97.5% (whiskers). **(c)** Mean absolute error (MAE) of daily model predictions with respect to the daily observed data, on the overall period (March 2020 – May 2022) and broken down by epidemic phase (epidemic waves and in-between periods). Dots and lines represent the average MAE and 95% confidence interval computed across n=100 stochastic runs.

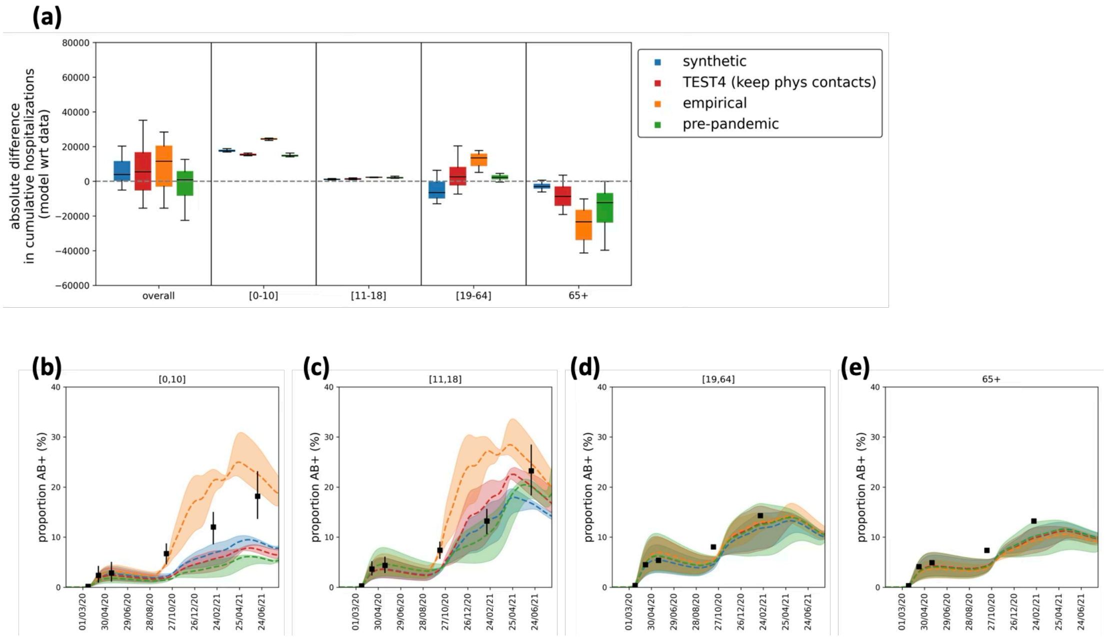

Figure S30. Sensitivity of age-stratified model outcomes using synthetic, empirical, pre-pandemic matrices, or matrices in TEST4. (a) Absolute difference (overall and by age class) of the cumulative number of hospital admissions (from March 1, 2020 to May 22, 2022) predicted in the four models (synthetic, TEST4, empirical and pre-pandemic), compared to observations. The box plot indicates median (line), interquartile range (box), and 2.5% and 97.5% quantiles (whiskers) out of n=100 independent stochastic runs. **(b-e)** Proportion of antibody-positive population over time, by age class (from left to right, [0,10], [11-18], [19-64], 65+). Black symbols indicate estimates from serological data. Lines and shaded areas indicate respectively the median and 95% probability ranges (2.5% and 97.5% quantiles) computed across n=100 independent stochastic simulations.

10. REFERENCES

1. Santé publique France. Données relatives aux personnes vaccinées contre la Covid-19 (VAC-SI). https://www.data.gouv.fr/fr/datasets/donnees-relatives-aux-personnes-vaccinees-contre-la-covid-19-1/ (2020).
2. Lauer, S. A. *et al.* The Incubation Period of Coronavirus Disease 2019 (COVID-19) From Publicly Reported Confirmed Cases: Estimation and Application. *Ann. Intern. Med.* **172**, 577–582 (2020).
3. Ferretti, L. *et al.* Quantifying SARS-CoV-2 transmission suggests epidemic control with digital contact tracing. *Science* **368**, eabb6936 (2020).
4. Lavezzo, E. *et al.* Suppression of a SARS-CoV-2 outbreak in the Italian municipality of Vo'. *Nature* **584**, 425–429 (2020).
5. Riccardo, F. *et al.* Epidemiological characteristics of COVID-19 cases and estimates of the reproductive numbers 1 month into the epidemic, Italy, 28 January to 31 March 2020. *Eurosurveillance* **25**, 2000790 (2020).
6. Davies, N. G. *et al.* Age-dependent effects in the transmission and control of COVID-19 epidemics. *Nat. Med.* **26**, 1205–1211 (2020).
7. Lapidus, N. *et al.* Do not neglect SARS-CoV-2 hospitalization and fatality risks in the middle-aged adult population. *Infect. Dis. Now* **51**, 380–382 (2021).
8. Cereda, D. *et al.* The early phase of the COVID-19 epidemic in Lombardy, Italy. *Epidemics* **37**, 100528 (2021).
9. Li, R. *et al.* Substantial undocumented infection facilitates the rapid dissemination of novel coronavirus (SARS-CoV2). *Science* https://doi.org/10.1126/science.abb3221 (2020) doi:10.1126/science.abb3221.
10. Hu, S. *et al.* Infectivity, susceptibility, and risk factors associated with SARS-CoV-2 transmission under intensive contact tracing in Hunan, China. *Nat. Commun.* **12**, 1533 (2021).
11. Franco, N. *et al.* Inferring age-specific differences in susceptibility to and infectiousness upon SARS-CoV-2 infection based on Belgian social contact data. *PLOS Comput. Biol.* **18**, e1009965 (2022).
12. Viner, R. M. *et al.* Susceptibility to SARS-CoV-2 Infection Among Children and Adolescents Compared With Adults: A Systematic Review and Meta-analysis. *JAMA Pediatr.* **175**, 143–156 (2021).
13. Boëlle, P.-Y. *et al.* Trajectories of Hospitalization in COVID-19 Patients: An Observational Study in France. *J. Clin. Med.* **9**, E3148 (2020).

14. Pellis, L. *et al.* Challenges in control of COVID-19: short doubling time and long delay to effect of interventions. *Philos. Trans. R. Soc. B Biol. Sci.* **376**, 20200264 (2021).

15. Hart, W. S. *et al.* Generation time of the alpha and delta SARS-CoV-2 variants: an epidemiological analysis. *Lancet Infect. Dis.* https://doi.org/10.1016/S1473-3099(22)00001-9 (2022) doi:10.1016/S1473-3099(22)00001-9.

16. Backer, J. A. *et al.* Shorter serial intervals in SARS-CoV-2 cases with Omicron BA.1 variant compared with Delta variant, the Netherlands, 13 to 26 December 2021. *Eurosurveillance* **27**, 2200042 (2022).

17. Gaymard, A. *et al.* Early assessment of diffusion and possible expansion of SARS-CoV-2 Lineage 20I/501Y.V1 (B.1.1.7, variant of concern 202012/01) in France, January to March 2021. *Eurosurveillance* **26**, 2100133 (2021).

18. Di Domenico, L., Goldberg, Y. & Colizza, V. Planning and adjusting the COVID-19 booster vaccination campaign to reduce disease burden. *Infect. Dis. Model.* **10**, 150–162 (2025).

19. Paredes, M. I. *et al.* Associations Between Severe Acute Respiratory Syndrome Coronavirus 2 (SARS-CoV-2) Variants and Risk of Coronavirus Disease 2019 (COVID-19) Hospitalization Among Confirmed Cases in Washington State: A Retrospective Cohort Study. *Clin. Infect. Dis.* ciac279 (2022) doi:10.1093/cid/ciac279.

20. Nyberg, T. *et al.* Comparative analysis of the risks of hospitalisation and death associated with SARS-CoV-2 omicron (B.1.1.529) and delta (B.1.617.2) variants in England: a cohort study. *The Lancet* https://doi.org/10.1016/S0140-6736(22)00462-7 (2022) doi:10.1016/S0140-6736(22)00462-7.

21. Pilz, S., Theiler-Schwetz, V., Trummer, C., Krause, R. & Ioannidis, J. P. A. SARS-CoV-2 reinfections: Overview of efficacy and duration of natural and hybrid immunity. *Environ. Res.* **209**, 112911 (2022).

22. Bastard, J. *et al.* Impact of the Omicron variant on SARS-CoV-2 reinfections in France, March 2021 to February 2022. *Eurosurveillance* **27**, 2200247 (2022).

23. Office for National Statistics. Coronavirus (COVID-19) Infection Survey, characteristics of people testing positive for COVID-19, UK - Office for National Statistics. https://www.ons.gov.uk/peoplepopulationandcommunity/healthandsocialcare/conditionsanddiseases/bulletins/coronaviruscovid19infectionsurveycharacteristicsofpeopletestingpositiveforcovid19uk/6october2021.

24. Altarawneh, H. N. *et al.* Protection against the Omicron Variant from Previous SARS-CoV-2 Infection. *N. Engl. J. Med.* https://doi.org/10.1056/NEJMc2200133 (2022) doi:10.1056/NEJMc2200133.

25. Suarez Castillo, M., Khaoua, H. & Courtejoie, N. Vaccine-induced and naturally-acquired protection against Omicron and Delta symptomatic infection and severe COVID-19 outcomes, France, December 2021 to January 2022. *Eurosurveillance* **27**, 2200250 (2022).
26. Carazo, S. *et al.* Protection against omicron (B.1.1.529) BA.2 reinfection conferred by primary omicron BA.1 or pre-omicron SARS-CoV-2 infection among health-care workers with and without mRNA vaccination: a test-negative case-control study. *Lancet Infect. Dis.* **23**, 45–55 (2023).
27. Chemaitelly, H. *et al.* Protection of Omicron sub-lineage infection against reinfection with another Omicron sub-lineage. *Nat. Commun.* **13**, 4675 (2022).
28. Santé publique France. Données relatives aux personnes vaccinées contre la Covid-19 (VAC-SI). https://www.data.gouv.fr/fr/datasets/donnees-relatives-aux-personnes-vaccinees-contre-la-covid-19-1/ (2020).
29. UK Health Security Agency. COVID-19 vaccine surveillance report: week 17.
30. Polack, F. P. *et al.* Safety and Efficacy of the BNT162b2 mRNA Covid-19 Vaccine. *N. Engl. J. Med.* **383**, 2603–2615 (2020).
31. Moreira, E. D. *et al.* Safety and Efficacy of a Third Dose of BNT162b2 Covid-19 Vaccine. *N. Engl. J. Med.* **386**, 1910–1921 (2022).
32. Tartof, S. Y. *et al.* Effectiveness of mRNA BNT162b2 COVID-19 vaccine up to 6 months in a large integrated health system in the USA: a retrospective cohort study. *The Lancet* **398**, 1407–1416 (2021).
33. Veneti, L. *et al.* Effectiveness of BNT162b2 vaccine against SARS-CoV-2 Delta and Omicron infection in adolescents, Norway, August 2021 to January 2022. *Int. J. Infect. Dis.* **130**, 182–188 (2023).
34. Menegale, F. *et al.* Evaluation of Waning of SARS-CoV-2 Vaccine–Induced Immunity: A Systematic Review and Meta-analysis. *JAMA Netw. Open* **6**, e2310650 (2023).
35. Dagan, N. *et al.* BNT162b2 mRNA Covid-19 Vaccine in a Nationwide Mass Vaccination Setting. *N. Engl. J. Med.* **384**, 1412–1423 (2021).
36. Haas, E. J. *et al.* Impact and effectiveness of mRNA BNT162b2 vaccine against SARS-CoV-2 infections and COVID-19 cases, hospitalisations, and deaths following a nationwide vaccination campaign in Israel: an observational study using national surveillance data. *The Lancet* **397**, 1819–1829 (2021).
37. Sheikh, A., McMenamin, J., Taylor, B. & Robertson, C. SARS-CoV-2 Delta VOC in Scotland: demographics, risk of hospital admission, and vaccine effectiveness. *The Lancet* **397**, 2461–2462 (2021).

38. Andrews, N. *et al.* Covid-19 Vaccine Effectiveness against the Omicron (B.1.1.529) Variant. *N. Engl. J. Med.* https://doi.org/10.1056/NEJMoa2119451 (2022) doi:10.1056/NEJMoa2119451.
39. UK Health Security Agency. COVID-19 vaccine surveillance report - week 6.
40. Collie, S., Champion, J., Moultrie, H., Bekker, L.-G. & Gray, G. Effectiveness of BNT162b2 Vaccine against Omicron Variant in South Africa. *N. Engl. J. Med.* **386**, 494–496 (2022).
41. Bobrovitz, N. *et al.* Protective effectiveness of previous SARS-CoV-2 infection and hybrid immunity against the omicron variant and severe disease: a systematic review and meta-regression. *Lancet Infect. Dis.* https://doi.org/10.1016/S1473-3099(22)00801-5 (2023) doi:10.1016/S1473-3099(22)00801-5.
42. Eyre, D. W. *et al.* Effect of Covid-19 Vaccination on Transmission of Alpha and Delta Variants. *N. Engl. J. Med.* https://doi.org/10.1056/NEJMoa2116597 (2022) doi:10.1056/NEJMoa2116597.
43. Pouwels, K. B. *et al.* Effect of Delta variant on viral burden and vaccine effectiveness against new SARS-CoV-2 infections in the UK. *Nat. Med.* **27**, 2127–2135 (2021).
44. Tan, S. T. *et al.* Infectiousness of SARS-CoV-2 breakthrough infections and reinfections during the Omicron wave. *Nat. Med.* 1–8 (2023) doi:10.1038/s41591-022-02138-x.
45. Altarawneh, H. N. *et al.* Effects of Previous Infection and Vaccination on Symptomatic Omicron Infections. *N. Engl. J. Med.* https://doi.org/10.1056/NEJMoa2203965 (2022) doi:10.1056/NEJMoa2203965.
46. French Ministry of Education. Déconfinement phase 2 : point de situation au 28 mai. *Ministère de l'Education Nationale et de la Jeunesse* https://www.education.gouv.fr/deconfinement-phase-2-point-de-situation-au-28-mai-303813 (2020).
47. French Ministry of Education. Calendrier scolaire. https://www.education.gouv.fr/calendrier-scolaire-100148 (2020).
48. Arregui, S., Aleta, A., Sanz, J. & Moreno, Y. Projecting social contact matrices to different demographic structures. *PLOS Comput. Biol.* **14**, e1006638 (2018).
49. Hu, H., Nigmatulina, K. & Eckhoff, P. The scaling of contact rates with population density for the infectious disease models. *Math. Biosci.* **244**, 125–134 (2013).
50. OECD. LFS by sex and age - indicators. https://stats.oecd.org/Index.aspx?DataSetCode=LFS_SEXAGE_I_R.
51. Santé publique France. CoviPrev : une enquête pour suivre l'évolution des comportements et de la santé mentale pendant l'épidémie de COVID-19. https://www.santepubliquefrance.fr/etudes-et-

enquetes/coviprev-une-enquete-pour-suivre-l-evolution-des-comportements-et-de-la-sante-mentale-pendant-l-epidemie-de-covid-19 (2020).

52. Pullano, G., Valdano, E., Scarpa, N., Rubrichi, S. & Colizza, V. Evaluating the effect of demographic factors, socioeconomic factors, and risk aversion on mobility during the COVID-19 epidemic in France under lockdown: a population-based study. *Lancet Digit. Health* **2**, e638–e649 (2020).
53. Van Kerckhove, K., Hens, N., Edmunds, W. J. & Eames, K. T. D. The Impact of Illness on Social Networks: Implications for Transmission and Control of Influenza. *Am. J. Epidemiol.* **178**, 1655–1662 (2013).
54. Di Domenico, L., Pullano, G., Sabbatini, C. E., Boëlle, P.-Y. & Colizza, V. Impact of lockdown on COVID-19 epidemic in Île-de-France and possible exit strategies. *BMC Med.* **18**, 240 (2020).
55. Pullano, G. *et al.* Underdetection of cases of COVID-19 in France threatens epidemic control. *Nature* **590**, 134–139 (2021).
56. Génois, M. & Barrat, A. Can co-location be used as a proxy for face-to-face contacts? *EPJ Data Sci.* **7**, 1–18 (2018).
57. Farrington, C. P., Whitaker, H. J., Wallinga, J. & Manfredi, P. Measures of Disassortativeness and their Application to Directly Transmitted Infections. *Biom. J.* **51**, 387–407 (2009).
58. Di Domenico, L. *Data-Driven Modeling of COVID-19 Spread in France to Inform Pandemic Response*. (2022).
59. Di Domenico, L. *et al.* Adherence and sustainability of interventions informing optimal control against the COVID-19 pandemic. *Commun. Med.* **1**, 1–13 (2021).
60. Santé publique France. Données hospitalières relatives à l'épidémie de COVID-19. https://www.data.gouv.fr/fr/datasets/donnees-hospitalieres-relatives-a-lepidemie-de-covid-19/ (2020).
61. Gimma, A., Wong, K. L., Coletti, P. & Jarvis, C. I. CoMix social contact data (France). Zenodo https://doi.org/10.5281/zenodo.6362893 (2021).
62. Funk, S. Introduction to socialmixr - Participants weights. https://cran.r-project.org/web/packages/socialmixr/vignettes/socialmixr.html#participant-weights.
63. Le Vu, S. *et al.* Prevalence of SARS-CoV-2 antibodies in France: results from nationwide serological surveillance. *Nat. Commun.* **12**, 3025 (2021).
64. Kelley, K. N. *et al.* Towards real-time monitoring of social contacts via participatory disease surveillance.
