# Supplementary material for: Mobility-driven synthetic contact matrices as a scalable solution for real-time pandemic response modeling
Source: Nat Commun. 2026 Jan 27;17:1845. doi: 10.1038/s41467-026-68557-3 (PMC12920812; doi:10.1038/s41467-026-68557-3)
Supplement: Supplementary file 2 — Reporting Summary [file 41467_2026_68557_MOESM2_ESM.pdf]

nature portfolio

Corresponding author(s): Vittoria Colizza

Last updated by author(s): Dec 19, 2025

Reporting Summary

Nature Portfolio wishes to improve the reproducibility of the work that we publish. This form provides structure for consistency and transparency in reporting. For further information on Nature Portfolio policies, see our Editorial Policies and the Editorial Policy Checklist.

Statistics

For all statistical analyses, confirm that the following items are present in the figure legend, table legend, main text, or Methods section.

n/a	Confirmed	
☐	☒	The exact sample size (*n*) for each experimental group/condition, given as a discrete number and unit of measurement
☒	☐	A statement on whether measurements were taken from distinct samples or whether the same sample was measured repeatedly
☐	☒	The statistical test(s) used AND whether they are one- or two-sided<br>*Only common tests should be described solely by name; describe more complex techniques in the Methods section.*
☐	☒	A description of all covariates tested
☒	☐	A description of any assumptions or corrections, such as tests of normality and adjustment for multiple comparisons
☐	☒	A full description of the statistical parameters including central tendency (e.g. means) or other basic estimates (e.g. regression coefficient) AND variation (e.g. standard deviation) or associated estimates of uncertainty (e.g. confidence intervals)
☐	☒	For null hypothesis testing, the test statistic (e.g. *F*, *t*, *r*) with confidence intervals, effect sizes, degrees of freedom and *P* value noted<br>*Give P values as exact values whenever suitable.*
☒	☐	For Bayesian analysis, information on the choice of priors and Markov chain Monte Carlo settings
☒	☐	For hierarchical and complex designs, identification of the appropriate level for tests and full reporting of outcomes
☐	☒	Estimates of effect sizes (e.g. Cohen's *d*, Pearson's *r*), indicating how they were calculated

Our web collection on statistics for biologists contains articles on many of the points above.

Software and code

Policy information about availability of computer code

Data collection	Not applicable as no data was collected
Data analysis	Analyses were carried out in Python 3.8.18. Code of the transmission model is publicly available at https://github.com/EPIcx-lab/COVID-19/tree/master/mobility_driven_synthetic_contact_matrices.

For manuscripts utilizing custom algorithms or software that are central to the research but not yet described in published literature, software must be made available to editors and reviewers. We strongly encourage code deposition in a community repository (e.g. GitHub). See the Nature Portfolio guidelines for submitting code & software for further information.

Data

Policy information about availability of data

All manuscripts must include a data availability statement. This statement should provide the following information, where applicable:

- Accession codes, unique identifiers, or web links for publicly available datasets
- A description of any restrictions on data availability
- For clinical datasets or third party data, please ensure that the statement adheres to our policy

Mobility-driven synthetic contact matrices and SocialCov contact matrices are available at https://github.com/EPIcx-lab/COVID-19/tree/master/mobility_driven_synthetic_contact_matrices. All other data used in the analyses are available online at the cited references.

Research involving human participants, their data, or biological material

Policy information about studies with human participants or human data. See also policy information about sex, gender (identity/presentation), and sexual orientation and race, ethnicity and racism.

Reporting on sex and gender	N/A
Reporting on race, ethnicity, or other socially relevant groupings	N/A
Population characteristics	We used age to stratify the French population in 4 age classes: children [0-10], adolescents [11-18], adults [19-64] and seniors with 65+ years old.
Recruitment	Hospitalization data stratified by age are available from https://www.data.gouv.fr/datasets/donnees-hospitalieres-relatives-a-lepidemie-de-covid-19/. Data were collected for surveillance purposes and we accessed aggregated anonymized data. No ethical approval was therefore required for this study.<br><br>This study used SocialCov human data that were collected and made available before the initiation of the study. SocialCov recruited participants through convenience sampling through the governmental app TousAntiCovid. In particular, the survey was promoted via the news channel of the app which invited individuals aged 18 and above to complete the questionnaire. Since the survey sample was not representative of the French population, synthetic populations were generated for each campaign to better reflect the age and gender distribution in France, using sampling with replacement from the SocialCov participant pool. PARTICIPANTS PROVIDED THEIR INFORMED CONSENT.
Ethics oversight	No ethical approval was required for this study.

Note that full information on the approval of the study protocol must also be provided in the manuscript.

Field-specific reporting

Please select the one below that is the best fit for your research. If you are not sure, read the appropriate sections before making your selection.

☒ Life sciences ☐ Behavioural & social sciences ☐ Ecological, evolutionary & environmental sciences

For a reference copy of the document with all sections, see nature.com/documents/nr-reporting-summary-flat.pdf

Life sciences study design

All studies must disclose on these points even when the disclosure is negative.

Sample size	We did not perform sample size calculations. We used n=100 independent stochastic simulation of the transmission model, to derive summary statistics of the epidemic trajectory, i.e. median and 95% probability ranges. This sample size was chosen as it provided stable summary statistics compared with other independent sets of simulations of the same sample size.
Data exclusions	We considered the contact matrix estimated for a regular weekday, defined as Monday-Friday excluding holidays. We did not model explicitly the weekday/weekend effect, which is absorbed in the calibration procedure of the transmission model (see section Inference Framework).
Replication	Each stochastic realization of the transmission model was independently executed, by assigning a distinct seed to initialize the random number generator, and can be replicated using the same seed. We derived summary statistics (i.e. median and 95% probability ranges) from 100 independent stochastic runs. The summary statistics gave stable results when applied to independent sets of stochastic simulations of the same sample size.
Randomization	Our study is observational and involves no intervention. Randomization of samples is therefore not applicable.
Blinding	Our study is observational and involves no intervention. Blinding techniques are therefore not applicable.

Reporting for specific materials, systems and methods

We require information from authors about some types of materials, experimental systems and methods used in many studies. Here, indicate whether each material, system or method listed is relevant to your study. If you are not sure if a list item applies to your research, read the appropriate section before selecting a response.

Materials & experimental systems

n/a	Involved in the study
☒	☐ Antibodies
☒	☐ Eukaryotic cell lines
☒	☐ Palaeontology and archaeology
☒	☐ Animals and other organisms
☒	☐ Clinical data
☒	☐ Dual use research of concern
☒	☐ Plants

Methods

n/a	Involved in the study
☒	☐ ChIP-seq
☒	☐ Flow cytometry
☒	☐ MRI-based neuroimaging

Plants

Seed stocks

Report on the source of all seed stocks or other plant material used. If applicable, state the seed stock centre and catalogue number. If plant specimens were collected from the field, describe the collection location, date and sampling procedures.

Novel plant genotypes

Describe the methods by which all novel plant genotypes were produced. This includes those generated by transgenic approaches, gene editing, chemical/radiation-based mutagenesis and hybridization. For transgenic lines, describe the transformation method, the number of independent lines analyzed and the generation upon which experiments were performed. For gene-edited lines, describe the editor used, the endogenous sequence targeted for editing, the targeting guide RNA sequence (if applicable) and how the editor was applied.

Authentication

Describe any authentication procedures for each seed stock used or novel genotype generated. Describe any experiments used to assess the effect of a mutation and, where applicable, how potential secondary effects (e.g. second site T-DNA insertions, mosiacism, off-target gene editing) were examined.
